# Blocks of Identity by Descent Reveal the Dual Role Played by Reef Fish Larvae for Retention and Dispersal

**DOI:** 10.1111/mec.70569

**Published:** 2026-09-28

**Authors:** Nina Tombers, Francisco Campuzano‐Jiménez, Martin Helmkampf, Janina Fuß, W. Owen McMillan, Oscar Puebla

**Affiliations:** ^1^ Leibniz Center for Tropical Marine Research Bremen Germany; ^2^ Institute for Chemistry and Biology of the Marine Environment (ICBM) Oldenburg Germany; ^3^ Stuttgart State Museum of Natural History Stuttgart Germany; ^4^ University of Antwerp Antwerpen Belgium; ^5^ Institute of Clinical Molecular Biology Kiel University Kiel Germany; ^6^ Smithsonian Tropical Research Institute (STRI) Apartado Panama Republic of Panama

**Keywords:** blocks of identity by descent, demographic inference, dispersal, *Hypoplectrus*, isolation by distance, reef fishes

## Abstract

Isolation by distance, whereby genetic distance between individuals or populations increases with geographic distance, is a fundamental population genetic pattern that is observed across a broad range of taxa and spatial scales. This pattern can be driven by dispersal limitation and be thereby informative about dispersal rate (σ), a parameter of both basic and applied interest. Yet isolation by distance is typically directly estimated from microsatellite or single nucleotide polymorphism data, which does not permit the direct estimation of σ or allow to disentangle dispersal limitation from other processes. Blocks of identity by descent (IBD) provide another lens through which isolation by distance may be viewed, and the opportunity to overcome these limitations. Here, we apply this framework to the barred hamlet ( *Hypoplectrus puella* ), a reef fish with a 3‐week planktonic larval phase. We consider two spatial scales, a continuous stretch of 200 km of reef along the Mesoamerican Barrier Reef in Belize and the Greater Caribbean across > 3000 km. The isolation by distance patterns show a good fit with theoretical expectations in Belize, and provide a recent σ estimate of 31 $\mathrm{km}/\sqrt{\mathrm{gen}}$ (95% confidence interval 23–40). The data also show a good fit with theoretical expectations at the Greater Caribbean scale, which is remarkable given the patchy nature of coral reef habitat at this scale, and provide a recent σ estimate of 248 $\mathrm{km}/\sqrt{\mathrm{gen}}$ (95% confidence interval 231–265). These results highlight the dual role played by coral reef fish larvae, which mediate both local retention and long‐distance dispersal.

## 1 Introduction

The notion of isolation by distance goes back to Wright (1938, 1940, 1943). Wright's insight was that in a continuously distributed population in which dispersal is limited, genetic differentiation between subpopulations is expected to increase with geographic distance. Based on an analytical model, he derived predictions for the expected increase in genetic differentiation among populations distributed in one and two dimensions. This seminal work was followed by Malécot (1948), Kimura (1953) and others, who built on Wright's model and extended it to the cases of discrete populations and differentiation among individuals.

Wright suggested that isolation by distance ‘may be of considerable importance’ (Wright 1940), but population genetic data were lacking at the time to test his model. With the advent of empirical population genetics, isolation by distance has been shown across a broad range of taxa and spatial scales, in continuous and discrete populations, in one and two dimensions, and at the population and individual levels (Friedlaender 1971; Palumbi et al. 1997; Wirth and Bernatchez 2001; Watts et al. 2007; Vos and Velicer 2008; Aguillon et al. 2017; Twyford et al. 2020; Benestan et al. 2021). Today it is generally appreciated that isolation by distance is a fundamental population genetic concept with broad theoretical and empirical relevance.

An important aspect of isolation by distance is its link with dispersal limitation. Isolation by distance is not expected in a panmictic population or in an island model of population structure where dispersal is spatially unrestricted (Wright 1931, 1940). Thus, isolation by distance is often interpreted as a population genetic ‘signature’ of dispersal limitation. What is more, the extent of isolation by distance, that is, the slope of the linear regression between geographic and genetic distance, may be informative about the spatial scale of dispersal. This was notably formalised by Rousset, who derived analytical approximations that explicitly link the slope of the isolation by distance regression among populations (Rousset 1997) and individuals (Rousset 2000) to $D_{e}\sigma^{2}$, the product of effective population density ($D_{e}$) and dispersal rate ($\sigma^{2}$) in one and two dimensions. These results provide the opportunity to estimate σ from isolation by distance, which is broadly relevant since isolation by distance is routinely detected with population genetic markers and σ is a parameter of both basic and applied interest. Nevertheless, such inference requires an *ad hoc* estimate of $D_{e}$ to extract σ from $D_{e}\sigma^{2}$, which is notoriously challenging, particularly for large and/or discontinuous populations. Furthermore, this approach assumes equilibrium between gene flow, mutation and genetic drift, which may often not be the case in marine populations characterised by large effective population sizes. Finally, this approach does not allow to disentangle recent dispersal limitation from ancestral dispersal limitation as well as other mechanisms such as colonisation, adaptation to environmental gradients or admixture gradients that may also generate a signal of isolation by distance (Meirmans 2012; Duranton et al. 2019).

Blocks of identity by descent (IBD) provide another lens through which isolation by distance may be viewed. IBD blocks are continuous and homologous tracts of DNA that are co‐inherited from a common ancestor. They tend to be broken up by recombination every generation, which provides the opportunity to derive expectations for their age given their length. This property is particularly useful for demographic inference (Palamara et al. 2012; Palamara and Pe'er 2013; Harris and Nielsen 2013; Browning et al. 2018), including in isolation by distance contexts (Baharian et al. 2016; Al‐Asadi et al. 2019). In particular, Ringbauer et al. (2017) derived the expected number of shared IBD blocks of length L in a population with constant size distributed in two dimensions:

(1) $$E\left[N_{L}\right]=\frac{G}{8\mathrm{\pi D}\sigma^{2}}{\left(\frac{r}{\sqrt{L}\sigma }\right)}^{2}K_{2}\left(\sqrt{2L}\frac{r}{\sigma }\right),$$

where G is total map length in Morgans, r geographic distance and $K_{2}$ the modified Bessel function of the second kind of degree two. This result implies an exponential decay of shared IBD blocks with geographic distance, which becomes more pronounced with increasing block length and increasing geographic distance. Ringbauer et al. (2017) also developed an inference scheme that allows to jointly estimate recent σ and $D_{e}$, which constitutes a significant methodological improvement over classic inference approaches based on isolation by distance, and applied it to human populations from Eastern Europe. More recently, Campuzano‐Jiménez et al. (2026) developed a Julia package that implements the method efficiently, which provides the opportunity to apply it broadly, including in non‐model organisms. This is what we do here, using the barred hamlet ( *Hypoplectrus puella* ) as a case study.

The hamlets are reef fishes from the Greater Caribbean that are known for their exceptionally high diversification rate (McCartney et al. 2003; Hench et al. 2022; Helmkampf et al. 2025). The genus includes 19 described species (Puebla et al. 2022, 2025). The barred hamlet is the most widespread and abundant hamlet species, and the only one that is also found in Bermuda. Juveniles and adults remain associated with coral reef habitat, but a three‐week planktonic larval phase provides potential for long‐distance dispersal with marine currents. A population genetic study of this species based on 10 microsatellite markers demonstrated isolation by distance at spatial scales ranging from 175 km in Guna Yala (Panama) to > 3500 km across the Caribbean Sea (Puebla et al. 2009). Considering the smallest spatial scale of 175 km, isolation by distance provided a σ estimate of 17 km/generation. However, this estimate relied on an *ad hoc* estimate of $D_{e}$ derived from the same microsatellite data, and the upper confidence interval was unbounded due to the small isolation by distance slope. The emergence of isolation by distance at a spatial scale of just 175 km in *H. puella* indicated that it is relevant to consider such a local scale for the study of dispersal, and that this would be most efficiently done by working at the individual level in continuous populations of the type envisioned by Wright.

Here, we leverage the hamlet linkage map (Theodosiou et al. 2016), reference genome (Hench et al. 2019), resequencing data from the Greater Caribbean (Puebla et al. 2009; Coulmance et al. 2024) and tissue samples from Belize (Puebla et al. 2012) to revisit isolation by distance in *H. puella* using IBD blocks and the framework developed by Ringbauer et al. (2017). In particular, we aim to assess whether the *H. puella* data fit theoretical expectations, and if so to jointly infer recent σ and $D_{e}$ from this signal. Since the Ringbauer et al. (2017) approach assumes a spatially homogeneous population, we expect a better fit with empirical data in Belize, where coral reef habitat is continuous, than at the Greater Caribbean scale where coral reef habitat is patchy and represents a just small fraction of the seafloor.

## 2 Methods

### 2.1 Sampling and Field Surveys

We considered two spatial scales, a continuous stretch of 200 km of reef along the Mesoamerican Barrier Reef in Belize (local scale) and > 3000 km across the Greater Caribbean. Samples for the local scale analysis were collected in June and July 2010 as detailed in Puebla et al. (2012). Briefly, 85 sampling sites separated by 2.5 km were considered on the seaward side of the Mesoamerican Barrier Reef in Belize (Figure 1). A single *H. puella* individual was targeted per sampling site, at depths ranging between 3 and 30 m. In addition, a 400 m^2^ transect survey was conducted at every other sampling site to evaluate the census density of *H. puella* in the sampling area as detailed in Puebla et al. (2012). Two scuba divers swam along a 100 m measuring tape and counted all individuals within two meters on each side of the tape. The surveys were conducted during the day at depths ranging between 5 and 20 m (mean 11 m). The position of each survey was chosen randomly, but avoiding areas without coral such as bare sand. Accordingly, the surveys covered a variety of reef habitats that include fore reef, spur‐and‐groove and reef wall.

**FIGURE 1:**
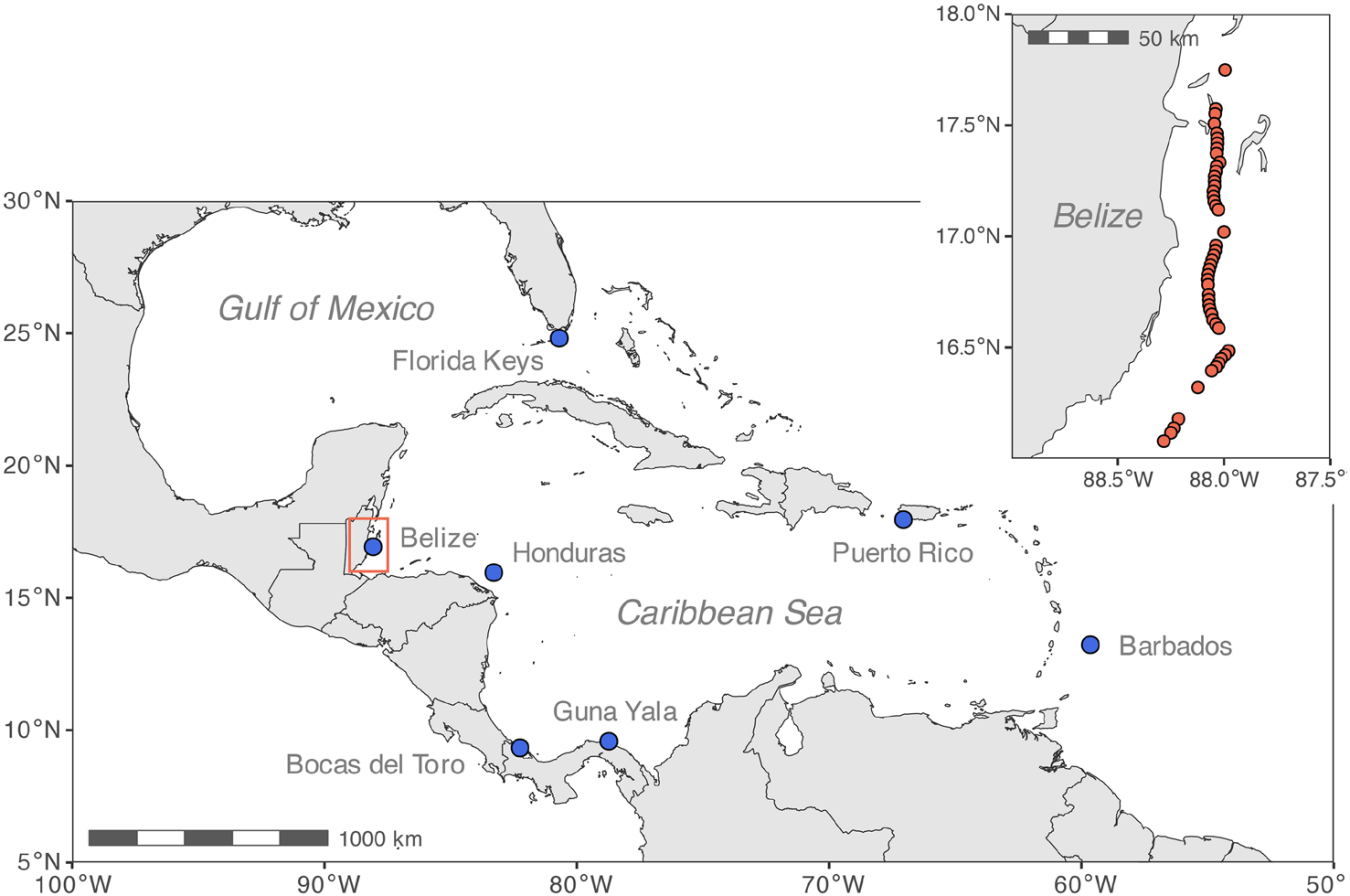
Sampling design. Two spatial scales were considered. At the local scale, individuals were sampled following a 200‐km transect along the Belize Barrier Reef in Belize, aiming at one individual every 2.5 km (inset; red dots). At the Greater Caribbean scale, samples were collected at six additional locations across the Caribbean Sea (blue dots).

Samples for the Greater Caribbean scale analysis were collected in the Florida Keys, Cayos Becerros (Honduras), Bocas del Toro (Panama), Guna Yala (Panama), La Parguera (Puerto Rico) and Barbados (Figure 1) between May 2004 and March 2017 as detailed in Puebla et al. (2009) and Coulmance et al. (2024). Temporal genetic structure is minimal in the barred hamlet (Puebla et al. 2009) and is therefore not expected to confound spatial genetic structure. Temporal genetic variation over 13 years is also not expected to be an issue for the range of IBD block lengths detected in this study, which are likely older than that. We considered a maximum of 10 samples per site to limit differences in sample size among sites, and to end up with a total sample size that is comparable to the local scale analysis. For Belize we randomly selected 10 samples from the Belize Barrier Reef samples.

Samples were collected on scuba with microspears or hook‐and‐line, either as fin‐clip samples or voucher specimens. Fieldwork was conducted under research permits 000017‐10 (Belize), NOAA ONMS 2017‐042 and FWCC SAL‐17‐1890A‐SR (Florida), 09/05/16 (Honduras), DAEX‐94 (Panama), #2016‐IC‐127 (E) (Puerto Rico) and 17/06/05 (Barbados), and Smithsonian Tropical Research Institute (STRI) Institutional Animal Care and Use Committee (IACUC) protocol 2017‐0101‐2020‐2.

### 2.2 Genotyping

The 49 samples for the local scale (Belize) analysis were sequenced anew for this study. Genomic DNA was extracted with Qiagen MagAttract High Molecular Weight kits. Illumina Nextera DNA Flex libraries were prepared and sequenced at the Institute of Clinical Molecular Biology (Kiel University) on an Illumina NovaSeq 6000. All 88 samples considered in this study were genotyped anew to produce two datasets, one for the local scale analysis and one for the Greater Caribbean. The variant calling procedure was based on the germline short variant discovery workflow and recommendations for GATK (McKenna et al. 2010) by the Broad Institute (DePristo et al. 2011; Van der Auwera et al. 2013). Briefly, raw reads were mapped to the hamlet reference genome (Hench et al. 2019) with BWA (Li and Durbin 2009) after adapter trimming. Duplicated reads were removed, and genotype likelihoods were calculated independently for each sample. Samples of both spatial scales were then genotyped jointly based on the individual genotype likelihoods. The dataset was restricted to variant sites meeting strict thresholds regarding read depth, mapping quality and mapping biases (i.e., sites with MQ < 57.5, QD < 3.0, FS > 60.0, MQRankSum < −0.2 || > 0.2 and ReadPosRankSum < −2.25 || > 2.25 were removed). Only biallelic sites present in at least 90% of samples were retained using VCFtools v0.1.16 (Danecek et al. 2011). Finally, the 49 samples from Belize were subset to produce the local‐scale dataset. For the Greater Caribbean‐scale dataset, only 10 randomly selected samples from Belize were retained, ensuring that both datasets comprised 49 samples.

### 2.3 Identification of IBD Blocks

Blocks of identity by descent were identified between all pairs of individuals from the SNP data with TRUFFLE (Dimitromanolakis et al. 2019). This approach was chosen because it does not rely on phasing, corrects for block break‐ups that are due to genotyping errors, and is computationally efficient. The minimum number of consecutive SNPs required to identify an IBD block was empirically set to 500 for IBD blocks that share one haplotype (IBD1) and to 250 for IBD blocks that share both haplotypes (IBD2).

In order to assess the robustness of the method to the IBD calling method, IBD blocks were also identified with the templated positional Burrows‐Wheeler transform (TPBWT, Freyman et al. 2021) at the local scale. This approach requires phasing but corrects for phase switch errors (as well as genotyping errors), and is also computationally efficient. The SNP data were phased with the read‐aware approach implemented in SHAPEIT2 (Delaneau, Howie, et al. 2013; Delaneau, Zagury, and Marchini 2013), and TPBWT was run with a minimum length for the identification of IBD blocks empirically set to 10,000 bp.

One issue with the consideration of IBD blocks in terms of number of SNPs or bp is that the Ringbauer et al. (2017) approach considers recombination explicitly, and IBD block lengths accordingly in cM, while physical lengths are confounded by recombination rates that vary along chromosomes. Specifically, longer IBD blocks are expected in low‐recombining regions. In addition, genomic regions under stabilising selection are expected to present high levels of sequence similarity (even though we have no a priori reason to think that stabilising selection is particularly strong in *H. puella* and did not attempt to identify it directly as we are only interested in its potential effect on IBD sharing in the context of this study). Thus, low recombination rates and stabilising selection may contribute to an overestimate of IBD sharing. In order to limit these effects, physical lengths were converted to cM using the hamlet linkage map (Theodosiou et al. 2016). This was done by mapping the IBD blocks to the hamlet reference genome and retrieving region‐specific recombination rates in cM/Mb from the linkage map. Furthermore, the distribution of IBD blocks along chromosomes was assessed. As expected, this distribution varied markedly along chromosomes, following broadly the *Hypoplectrus* recombination landscape (Theodosiou et al. 2016; Hench et al. 2019, 2022), and presented sharp peaks (Figure S1A,B). A large region of chromosome 8 that had been previously identified as a putative inversion also showed high levels of IBD sharing. In an effort to reduce the effect of low recombination and stabilising selection, the IBD blocks that overlapped with regions of the genome above the 95th percentile of the genomic frequency of IBD blocks (Figure S1A,B) were filtered out. Furthermore, IBD blocks smaller than 0.4 cM were not considered to limit the occurrence of false positives.

### 2.4 Isolation by Distance

We started by assessing isolation by distance at both spatial scales using SNPs across the whole genome. For this we considered relatedness between pairs of individuals using the unadjusted $A_{\mathrm{jk}}$ statistic (Yang et al. 2010) as implemented in VCFtools (Danecek et al. 2011). We then considered all IBD blocks at both spatial scales to assess whether IBD blocks provide a similar pattern of isolation by distance. For this we used the following genome‐wide IBD index between pairs of individuals

(2) $$\frac{0.5\times \mathrm{IBD}1+\mathrm{IBD}2}{\mathrm{IBD}0+\mathrm{IBD}1+\mathrm{IBD}2},$$

where IBD0, IBD1 and IBD2 correspond to the summed lengths of all fragments that have 0, 1 and 2 haplotypes that are identical by descent, respectively, over the entire genome. At the local scale, distance between pairs of sampling sites (in multiples of 2.5 km that separate adjacent sampling sites) was used as geographic distances. At the Greater Caribbean scale, geographic distances between sampling locations were measured following the shortest in‐water path calculated with the R package marmap based on NOAA bathymetry data. Since the isolation by distance signal was subtle at the local scale, the analysis was repeated using the alternative method to identify IBD blocks (TPBWT) and without filtering IBD blocks to test its robustness.

### 2.5 Model Fit and Demographic Inference

We used IdentityByDescentDispersal.jl (Campuzano‐Jiménez et al. 2026) to fit the Ringbauer et al. (2017) model to our empirical data at both the local and Greater Caribbean scales. We considered three IBD block size bins (0.4–1 cM, 1–2.5 cM and 2.5–5 cM), which were empirically determined to cover the range of IBD block sizes in our dataset. We considered both a constant population density model and a power‐density model where population density grows at a fixed rate β.

The Ringbauer et al. (2017) model is explicitly two‐dimensional, while the topology of the Belize Barrier Reef may suggest that a one‐dimensional model would be more appropriate at the local scale. Yet the Belize Barrier Reef is just one part of the Mesoamerican Barrier Reef System that also includes shallow reefs along the coastal lagoon, deeper reefs further offshore, as well as islands and atolls, notably the large Turneffe, Lighthouse and Glovers atolls in the vicinity of our study area. The barred hamlet is present in all these habitats, and when they are considered, our sampling area in Belize is in the central part of a > 60 km wide habitat. The application of a two‐dimensional model is therefore justified.

Demographic inference, that is, the estimation of σ and $D_{e}$ from the IBD block data using the Ringbauer et al. (2017) framework, was done concurrently with IdentityByDescentDispersal.jl. Inference was also done at both spatial scales since contrary to our hypothesis the empirical data presented a good fit with theoretical expectations at both spatial scales. We considered IBD blocks > 1 cM because we were interested in estimating recent dispersal specifically, as this is most relevant from a management and conservation perspective. The inference was done using maximum‐likelihood estimation (MLE) following Ringbauer et al. (2017) as implemented in IdentityByDescentDispersal.jl.

In order to get a sense of the temporal scope of our estimates, we also used IdentityByDescentDispersal.jl to compute the expected age of IBD blocks across the range of geographic distances and block size length considered for demographic inference.

### 2.6 Validation

The Ringbauer et al. (2017) model assumes that haplotypes disperse following a Brownian motion across a continuous habitat represented by a two‐dimensional torus. This assumption is necessary to make the inference tractable, but is likely to be violated in natural populations. To validate the dispersal estimates, we tested the inference framework with simulated datasets where this assumption was relaxed. Following Chevy et al. (2025), we used SLiM 5 (Haller et al. 2026) to simulate a Wright‐Fisher population in a continuous space and msprime (Baumdicker et al. 2022) to perform recapitation (Haller et al. 2019). First, we relaxed the assumption that individuals are distributed across a two‐dimensional torus and considered instead a square area limited by borders. Furthemore, coral reefs in the Greater Caribbean are patchy and cover only a small fraction of the seafloor. To model this, we simulated a highly heterogeneous space with only 1% of the surface covered by patches of high habitat suitability (‘coral reefs’) and the remaining 99% corresponding to patches of low habitat suitability (to which we assigned a fitness reduction of 99% to account for the fact that barred hamlets are occasionally observed on non‐coral‐reef habitat). We modelled mating and subsequent dispersal separately and tracked mean dispersal across generations in each simulation. We assumed individuals tend to choose nearby mates and are under density‐selection (because resources are finite). Specifically, we modelled both mating and density‐selection with a Gaussian kernel with constant standard deviation of 0.01 distance units. Mean dispersal was increased by drawing new positions from a Gaussian kernel with increasing standard deviations. A total of 120 simulations across varying dispersal rates and number of individuals were conducted. IdentityByDescentDispersal.jl was then used to conduct demographic inference and compare the estimated parameters to the simulated ones.

The scripts used for data analysis and simulations are available at https://github.com/currocam/tombers_etal.

## 3 Results

### 3.1 Sampling, Field Surveys and Genotyping

*H. puella* samples could be collected at 52 of the 85 sampling sites in Belize, with a distance between pairs of samples ranging between 2.5 km for samples collected in adjacent sites and 200 km for the most distant pair of samples. Of these 52 samples, 49 were sequenced and all these genomes are new to this study (Figure 1, Table S1). For the Greater Caribbean scale analysis, a total of 49 genomes from seven locations (Hench et al. 2019; Coulmance et al. 2024; Helmkampf et al. 2025) were considered, with a sample size ranging between 3 and 10 samples per site (mean 7, Table S2). The distance between pairs of sites ranged between 388 and 3076 km at the Greater Caribbean scale.

A total of 43 field surveys were conducted at the sampling sites in Belize. Between zero and two *H. puella* individuals were observed per 400 m^2^ survey, providing a census density estimate of 698 ± 209 (standard error) individuals per km^2^ of reef. The barred hamlet density was relatively constant along the 212.5 km portion of the Belize Barrier Reef that was surveyed, with a slight tendency to increase towards the southern part (Figure S2). Thus, our local scale study design based on a continuous population sampled continuously at the individual level is as close as it gets to an ideal reef fish population, notwithstanding a few sampling gaps along the barrier reef.

The 88 genomes considered in this study provided a total of 13,932,149 SNPs. Mean sequencing depth per sample (averaged across sites) ranged from 16.3× to 37.9×, with a mean of 24.1× across samples after removing duplicate reads. The metadata, sequencing depth and accession numbers of all genomes considered in this study are provided in Tables S1 and S2.

### 3.2 IBD Blocks

At the local scale, TRUFFLE identified a total of 50,835 IBD blocks larger than 0.4 cM after 95th percentile filtering. This provided an average of 43.67 IBD blocks per pair of samples ranging between 0.4 and 19.02 cM (Figure S3A). At the Greater Caribbean scale, TRUFFLE identified a total of 39,725 IBD blocks larger than 0.4 cM after 95th percentile filtering. This provided an average of 34.13 IBD blocks per pair of samples ranging between 0.4 and 19.02 cM (Figure S3A). Thus, fewer IBD blocks were detected per pair at the Greater Caribbean scale than at the local scale, which is consistent with the expectation that individuals are less related at the Greater Caribbean scale than at the local scale.

The longest IBD block (19.02 cM) was an outlier that was repeatedly recovered on linkage group 14 for a variety of sample pairs at both spatial scales. It is therefore likely to be a false positive, yet IBD blocks > 5 cM were anyhow not considered in downstream analyses due to their rare occurrence.

TPBWT identified fewer IBD blocks than TRUFFLE in Belize (18,861 versus 50,835, respectively), but with a similar length distribution (Figure S3B). One notable difference was the lower relative proportion of the smallest bin class in the TRUFFLE dataset, which is consistent with the fact that TRUFFLE has low power to detect short IBD fragments (Dimitromanolakis et al. 2019). The distribution of the TPBWT IBD blocks along the genome was broadly similar to the TRUFFLE one, following the *Hypoplectrus* recombination landscape, but with small‐scale differences, notably the position of sharp peaks of occurrence (Figure S1C).

### 3.3 Isolation by Distance

As expected, the SNP data provided a subtle pattern of isolation by distance at the local scale in Belize (Figure 2A). A similar pattern was recovered with the IBD index (Figure 2B), as well as when not applying the 95th percentile filter and calling IBD blocks with TPBWT instead of TRUFFLE (Figure S4). This pattern is characterised by a small isolation by distance slope and a high scatter, which is typical at the individual level in continuous populations (Rousset 2000; Sumner et al. 2001; Broquet et al. 2006; Watts et al. 2007; Aguillon et al. 2017). It is also similar to the isolation by distance patterns revealed by microsatellite markers in five other reef fish species (including the closely related black hamlet) collected along the same transect in Belize, as well as computer simulations (Puebla et al. 2009).

**FIGURE 2:**
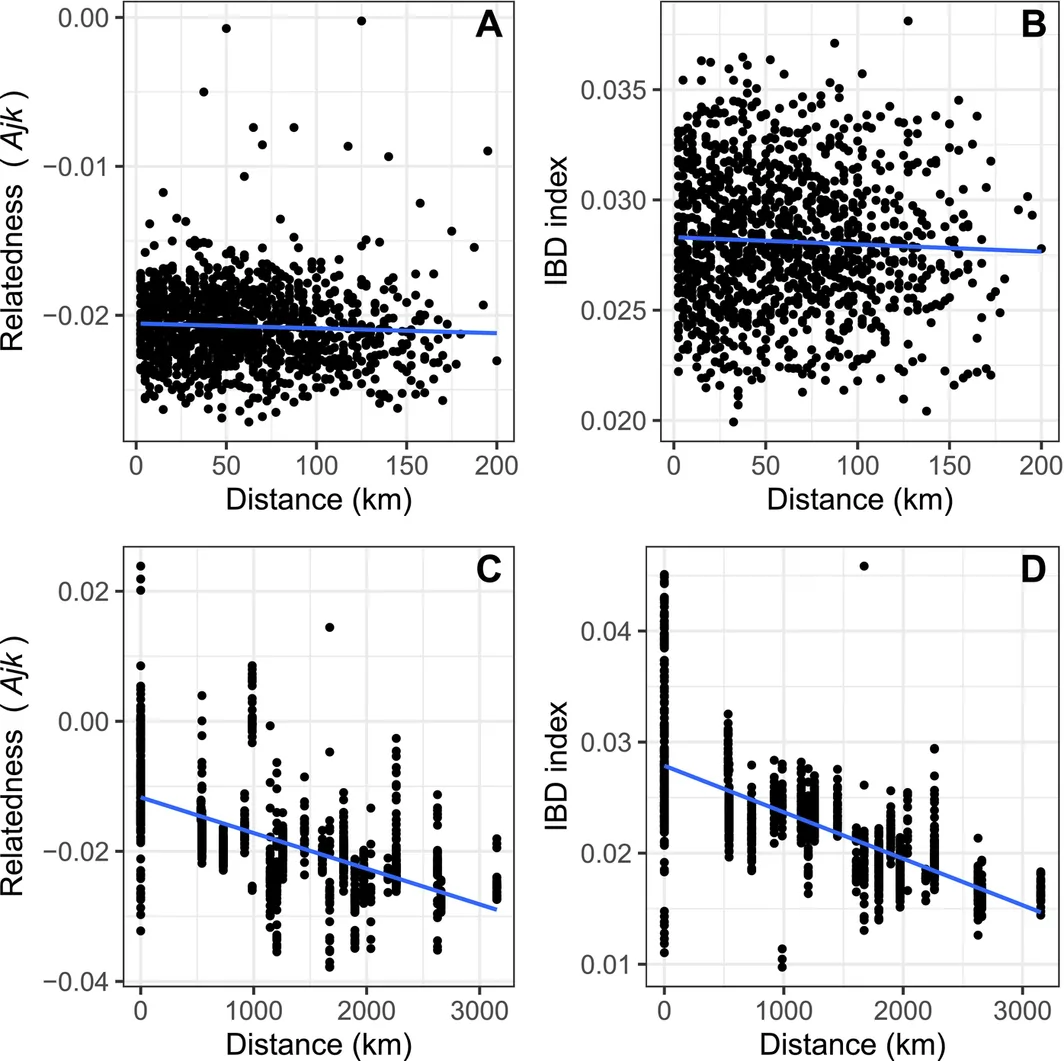
Isolation by distance between pairs of individuals at the local scale in Belize (A, B) and at the Greater Caribbean scale (C, D) based on pairwise relatedness from genome‐wide SNP data (A, C) and genome‐wide IBD index from all IBD blocks (B, D). As expected, a subtle pattern of isolation by distance is revealed at the local scale by the two approaches, and a stronger one at the Greater Caribbean scale. Note one pair of individuals that are > 1500 km apart at the Greater Caribbean scale but among the most closely related pairs with both approaches.

As expected again, the SNP data provided a clear pattern of isolation by distance at the Greater Caribbean scale, and a similar pattern was recovered with the IBD index (Figure 2C,D). This pattern was characterised by discrete geographic distance classes, which reflects the discrete sampling scheme at this scale (7 locations that provide 21 pairs of locations). In agreement with the local scale analysis, a high scatter was observed within sampling locations (geographic distance ≈0). Nevertheless, this scatter tended to decrease towards large geographic distances. This reflects the fact that a broad range of relatedness is observed within populations (pairs of individuals may be closely or distantly related), but the proportion of distantly related pairs increases with increasing geographic distance. Yet one pair of individuals that were sampled > 1500 km apart was among the most closely related pairs (Ajk > 0.01 and IBD index > 0.045). Both individuals were sampled in 2017, one in Puerto Rico and the other in the Florida Keys.

### 3.4 Model Fit and Demographic Inference

The isolation by distance patterns based on number of IBD blocks shared between pairs of individuals for three different IBD block size bins are presented in Figure 3. The error bars for the number of shared IBD blocks tended to increase with increasing block size, which is consistent with the fact that there are fewer long IBD blocks. The error bars for the number of shared IBD blocks also tended to increase with increasing geographic distance, which is consistent with the fact that there are fewer pairs of samples separated by large geographic distances. In agreement with theoretical expectations (Equation 1), the number of shared IBD blocks decreased exponentially with increasing geographic distance, and this decrease became more pronounced with increasing IBD block length and increasing geographic distance.

**FIGURE 3:**
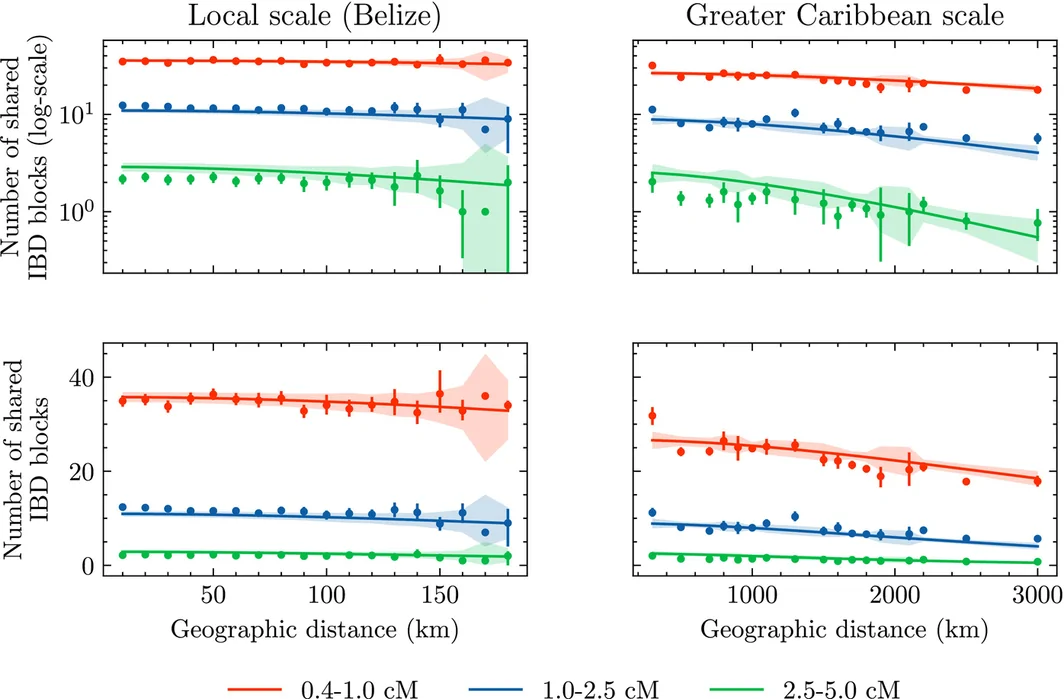
Isolation by distance based on IBD blocks at the local scale in Belize (left) and at the Greater Caribbean scale (right). The same data are presented on a logarithmic (top) and linear (bottom) scale. Three block size categories are considered: 0.4–1.0 cM (red), 1.0–2.5 cM (blue), and 2.5–5.0 cM (green). The dots represent the empirical data (binned, mean with their corresponding 95% bootstrapping confidence intervals), and the solid lines represent the maximum‐likelihood estimation (MLE) model fitted to the data. The shaded areas show the 95% confidence interval prediction under the model for the MLE. As expected, the number of shared IBD blocks decreases exponentially with geographic distance, and this decrease becomes more pronounced with increasing block length and increasing geographic distance.

The power density model provided the best fit with the empirical data (*β* = 0.641 and 0.554 for the local and Greater Caribbean scale, respectively), and the fit was generally good (Figure 3). One notable deviation was a larger number of short IBD blocks within populations (geographic distance ≈0) than predicted at the Greater Caribbean scale, which suggests that IBD of the most closely related pairs of individuals was overestimated (a similar pattern can be seen for humans from Eastern Europe in Ringbauer et al. (2017)). Other than that the fit was equally good at the local and Greater Caribbean scale, which was unexpected considering the patchy nature of coral reefs at the Greater Caribbean scale.

The results of the demographic inference are presented in Table 1. The model provided a recent σ estimate of 31 $\mathrm{km}/\sqrt{\mathrm{gen}}$ (95% confidence interval 23–40) at the local scale when considering IBD blocks > 1 cM. $D_{e}$ was estimated to 0.175 (95% confidence interval 0.090–0.250) individuals/km^2^. At the Greater Caribbean scale the model provided a recent σ estimate of 248 $\mathrm{km}/\sqrt{\mathrm{gen}}$ (95% confidence interval 231–265) and a $D_{e}$ estimate of 0.002 (95% confidence interval 0.002–0.003) individuals/km^2^. Figure 4 indicates that these estimates are expected to reflect dispersal limitation largely within the last 100 generations, which would represent at least 150 years since the *H. puella* reaches sexual maturity within a year and a half in captivity (T. Deuss, personal communication).

**TABLE 1:** Results of the demographic inference considering IBD blocks > 1 cM.

Spatial scale	Parameter	MLE	Lower 95% CI	Upper 95% CI
Local (Belize)	$D_{e}$	0.175	0.090	0.260
Local (Belize)	β	0.641	0.601	0.681
Local (Belize)	σ	31.4	23.3	39.6
Greater Caribbean	$D_{e}$	0.00250	0.00194	0.00306
Greater Caribbean	β	0.554	0.506	0.602
Greater Caribbean	σ	248.1	231.1	265.1

*Note:* σ in $\mathrm{km}/\sqrt{\mathrm{gen}}$, $D_{e}$ in individuals/km^2^.

Abbreviations: CI, confidence interval; MLE, maximum likelihood estimate.

**FIGURE 4:**
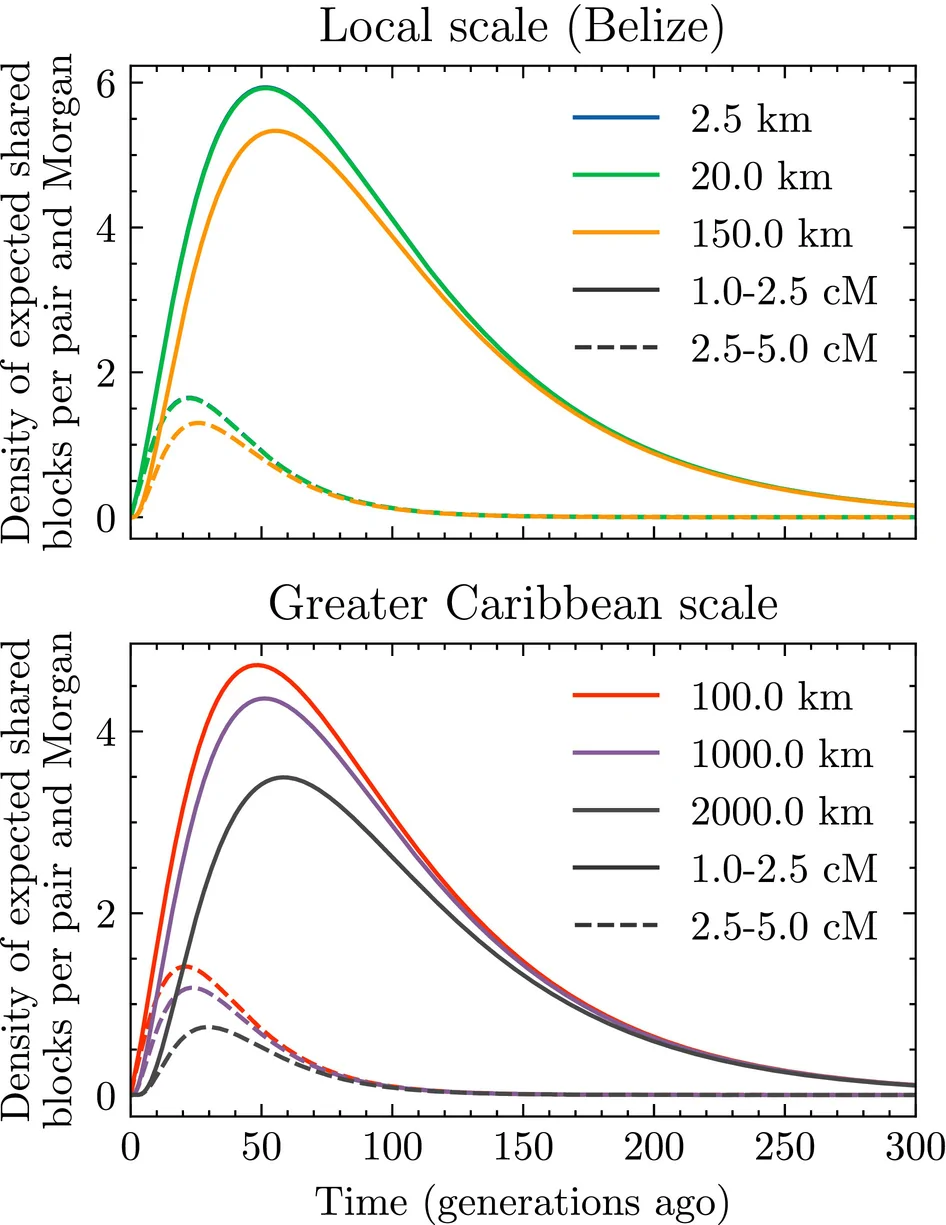
Expected age of the IBD blocks > 1 cM considered for σ and $D_{e}$ inference across a range of geographic distances at the local (top) and Greater Caribbean (bottom) scales. The inference applies largely to the recent past (< 100 generations).

In order to assess the consistency of the results across IBD calling methods, we also ran the inference at the local scale using the TPBWT data. The model provided a recent σ estimate of 26 $\mathrm{km}/\sqrt{\mathrm{gen}}$ (95% confidence interval 16–36), and $D_{e}$ was estimated to 0.338 individuals/km^2^ (95% confidence interval 0.089–0.587). This is broadly consistent with the results based on the TRUFFLE data, with overlapping 95% confidence intervals. The best fit was also with the power density model, and also provided a positive *β* estimate, albeit slightly lower than with the TRUFFLE data (*β* = 0.244, 95% confidence interval 0.148–0.340).

### 3.5 Validation

The dispersal estimates were generally robust to the model assumption violations that we tested. For high dispersal rates, the accuracy of the dispersal estimates increased with increasing spatial scale of sampling (Figure S5), which highlights the importance of sampling at large spatial scales to capture the effect of long‐dispersal events. Due to the simulation setup, the local density and $D_{e}$ estimates varied drastically depending on the dispersal rate. This is mainly because for individuals to occupy all suitable habitat patches, they first have to colonise them, which might not happen with high probability if dispersal rate is very low. This might result in a meta‐population with low connectivity and/or high local density, increasing the expected effective density and population size.

## 4 Discussion

To the best of our knowledge, this is the first study that applies the Ringbauer et al. (2017) approach to non‐human populations. The empirical data are consistent with the general predictions of the model. This includes, most notably, the observation of an exponential decrease of shared IBD blocks with increasing geographic distance, which becomes more pronounced with increasing IBD block length and increasing geographic distance. Beyond general trends, the model predictions also show a good fit with the empirical data, which is remarkable considering the technical challenges to identify IBD blocks from short‐read sequence data and to estimate their length in centimorgans.

At the local scale in Belize, IBD blocks provide a recent dispersal rate (σ) estimate of 31 $\mathrm{km}/\sqrt{\mathrm{gen}}$ (95% confidence interval 23–40) for the barred hamlet. This is higher than the σ estimate provided by microsatellite data at the local scale in Panama (17 km/generation), but this estimate had an unbounded upper 95% confidence interval (Puebla et al. 2009). IBD blocks allow therefore to refine this estimate by providing a finite upper 95% confidence interval, a timescale to which the estimate is expected to apply (mostly within the last 100 generations), and to jointly estimate effective density ($D_{e}$). A σ estimate in the order of 20–40 km is in line with evidence of local retention in reef fishes (Swearer et al. 1999; Jones et al. 1999; Almany et al. 2007), which may be explained by a combination of oceanographic factors and active larval retention mechanisms (Atema et al. 2015). Our dispersal rate estimate is also consistent with evidence of small‐scale connectivity among reef fish populations separated by a few tens of kilometres (Jones et al. 2022).

Small‐scale dispersal estimates derived from isolation by distance have been shown to be remarkably consistent with direct estimates of dispersal (Rousset 2000; Sumner et al. 2001; Broquet et al. 2006; Watts et al. 2007), including in reef fishes (Pinsky et al. 2017; Naaykens and D'Aloia 2022). It is nevertheless important to keep in mind that dispersal studies conducted at the local scale can by definition only detect local dispersal, leaving long‐distance dispersal—which may also be significant—uncharacterized.

The IBD data also show a good fit with the model at the Greater Caribbean scale, which is even more remarkable than at the local scale considering the patchy distribution of coral reef habitat at this spatial scale, and the fact that coral reefs represent just a small fraction of the Greater Caribbean. Furthermore, the simulations indicate that the dispersal estimates are quite robust with respect to habitat patchiness, particularly when sampling is conducted at a large spatial scale. Altogether, these two observations suggest that our dispersal estimates are likely to be robust not only at the local scale but also at the Greater Caribbean scale. This indicates that long‐distance dispersal is also substantial, and thereby also relevant for management and conservation (Manel et al. 2019). Isolation by distance has been evidenced before at large spatial scales in reef fishes, including in the barred hamlet (Puebla et al. 2009), but it was not straightforward to use this signal to infer dispersal due to the difficulty to independently estimate $D_{e}$ and disentangle recent dispersal from more ancestral processes at this spatial scale, as well a non‐equilibrium conditions between gene flow, mutation and genetic drift.

All in all, our local scale analysis provides evidence of local retention, our large‐scale analysis provides evidence of extensive dispersal, and we posit that the two are not in contradiction. They instead relate to different parts of the dispersal kernel, with the large‐scale analysis focusing on its tail. Long‐distance dispersal was further supported by high IBD between two individuals collected > 1500 km apart in the Florida Keys and Puerto Rico. Single nucleotide polymorphisms also indicated high relatedness between these two individuals, implying that this result is not an artefact of our IBD block detection approach. This result does not necessarily indicate a single > 1500 km dispersal event—the two individuals may be related through several dispersal events over several generations—but it is consistent with the occurrence of long‐distance dispersal events. Similarly, σ is best interpreted as an effective dispersal parameter that reflects the rate of the spatial spread of ancestry back in time rather than a single‐generation dispersal distance (Ringbauer et al. 2017). This may contribute to explain the contrast between the estimates at the two spatial scales. At the Greater Caribbean scale, IBD sharing between distant locations is expected to reflect recent historical dispersal events that effectively connect otherwise remote reefs, which may result in a filtering of the dispersal distance distribution compared with what is observed in a continuous habitat.

Blocks of identity by descent allow to travel in time by considering different block lengths, which provides insights for the interpretation of isolation by distance. In contrast to IBD blocks, microsatellite and SNP markers provide a picture of isolation by distance that integrates over all timescales, which may contribute to blur the signal when it is mostly due to recent dispersal limitation. This is indeed the case in the barred hamlet at the local scale, where one may question the reality of the isolation by distance signal provided by SNP markers due to the large scatter and small slope of the regression. The IBD index provides a similar pattern, but IBD blocks allow to dissect the signal into IBD block size classes. When this is done the picture becomes much clearer, with an increase of isolation by distance with increasing IBD block length and increasing geographic distance, which is consistent with theoretical expectations and therefore supports the view that this signal is real and driven by recent dispersal limitation. Such a situation may be relatively common since a subtle pattern of isolation by distance at the local scale similar to the one that we report here has been evidenced in a variety of taxa (Rousset 2000; Sumner et al. 2001; Broquet et al. 2006; Watts et al. 2007; Puebla et al. 2012; Aguillon et al. 2017).

Effective density was estimated to 0.175 (95% confidence interval 0.090–0.250) individuals/km^2^ in Belize, which is three orders of magnitude lower than the census density estimated from the field transects (698 ± 209 individuals/km^2^). This provides a $D_{e}/D$ ratio of 0.0003. This value is within the range of $N_{e}/N$ ratios reported in marine fishes (0.01–0.000001), which are likely due to high variance in reproductive success among individuals (Hauser and Carvalho 2008). The better fit with a power density model suggests a recent population expansion of *H. puella* . Independent demographic inference analyses are required to test this hypothesis.

The detection of IBD blocks is not straightforward, with over 20 methods developed to this aim (Browning and Browning 2012; Delaneau, Howie, et al. 2013; Delaneau, Zagury, and Marchini 2013; Park et al. 2015; Bjelland et al. 2017; Dimitromanolakis et al. 2019; Naseri et al. 2019; Zhou et al. 2020; Seidman et al. 2020; Freyman et al. 2021). The difficulty in calling IBD blocks is notably due to the occurrence of genotyping and phase switch errors. Computational requirements are also an issue when large panels of SNPs and individuals are considered, although this is arguably less of an issue for relatively small datasets like the ones considered here. The advent of next‐generation sequencing contributed to improving IBD blocks calling, but errors are still expected, particularly phase switch errors when genomes are not physically phased. This is notably the case for the TPBWT method that we implemented (which requires phasing), and one of the reasons that motivated us to use TRUFFLE (which does not require phasing) for our main analyses. Nevertheless, it is encouraging to see that the isolation by distance pattern and demographic inference in the barred hamlet is robust to filtering versus not filtering IBD blocks and using different IBD calling methods.

Furthermore, recombination is considered explicitly by the Ringbauer et al. (2017) approach and therefore considers IBD block lengths in cM, which requires a linkage map. One option that comes to mind in the absence of a linkage map would be to use an LD‐based population genetic approach to estimate recombination rate along the genome (Peñalba and Wolf 2020). Yet this approach typically provides population recombination rate $\rho =4N_{e}r$ where $N_{e}$ is effective population size and r recombination rate. A $N_{e}$ estimate would be needed to extract r from this product, which is not straightforward and would imply some circularity as effective density is precisely one of the parameters estimated by the inference. Thus, the requirement of a linkage map is difficult to circumvent. This may contribute to explain why the use of IBD blocks is still largely limited to the study of human populations. For marine fishes, the fact that females can produce a large number of eggs allows to generate a linkage map from a single cross, which is exactly what was done for the hamlets (Theodosiou et al. 2016). We note that some IBD calling methods allow to input a genetic map directly at the IBD calling stage, which is preferable to the two‐step procedure that we implemented, but may require the same set of markers for linkage mapping and IBD calling (which was not the case for this study).

Our approach relates to the study by Duranton et al. (2019), who estimated the spatial scale of dispersal in the European sea bass ( *Dicentrarchus labrax* ) using blocks of admixture. This approach is based on the premise that when a population receives genetic material from a distinct lineage, introgressed blocks tend to get shortened by recombination as they move away from the contact zone through multi‐generational dispersal. It has several parallels with the approach implemented here (it relies on the identification of haplotype blocks, does not assume equilibrium between gene flow, mutation and genetic drift, provides dispersal estimates that are independent of effective population size, …) and represents a valid alternative to our approach, critically so when gradients of admixture contribute to isolation by distance. Yet the Duranton et al. (2019) approach relies on the occurrence of a pulse of introgression as well as a spatial gradient of admixture, quite specific conditions that are also not straightforward to document in the first place. Furthermore, introgressed tracts may be under stronger selection than the rest of the genome, which would contribute to bias dispersal estimates.

### 4.1 Perspectives

Our study illustrates the potential of IBD blocks for demographic inference in non‐human populations. A major advantage of IBD blocks is that their length provides a timeline, from just one generation back in time for the largest IBD blocks up to thousands of generations for the shortest IBD blocks that may be identified with dense SNP data (Browning and Browning 2012). The analysis of IBD blocks will become more accessible as sequencing costs continue to drop and new inference methods such as the one implemented here are developed. Furthermore, the increasing accessibility to physically phased data, through either linked‐ or long‐read sequencing, as well as the continuous development of new approaches for the identification of IBD blocks, will contribute to improving the reliability of this approach. IBD blocks have a foot in the door of molecular ecology; they are here to stay and are likely to play a significant role in the field.

## Author Contributions

Nina Tombers contributed to the development of the project, performed all the analyses with the support of Martin Helmkampf and Francisco Campuzano‐Jiménez, and wrote the first draft of the manuscript. Martin Helmkampf contributed to the development of the project and to the data analysis. Francisco Campuzano‐Jiménez contributed to the analysis and conducted the simulations. Janina Fuß contributed to the sequencing. W. Owen McMillan contributed to the development of the project and to the fieldwork in Belize. Oscar Puebla developed the project, contributed to all the fieldwork, provided guidance and feedback throughout the analyses and contributed to the interpretation of the results and to the writing. All authors provided feedback on the manuscript.

## Funding

This study was funded by a German Research Foundation individual grant (number PU571/1–2) to OP and a National Geographic Committee for Research and Exploration grant (number 8799‐10) to OP. FC‐J was supported by the Research Foundation—Flanders (FWO) (grant FWO‐11A2P26N). Data analyses were performed at the HPC Cluster ROSA, located at the University of Oldenburg (Germany) and funded by the DFG through its Major Research Instrumentation Programme (INST 184/225‐1 FUGG) and the Ministry of Science and Culture (MWK) of Lower Saxony State.

## Ethics Statement

All the research complies with applicable laws on sampling from natural populations and animal experimentation (including the ARRIVE guidelines). This research was conducted under the Smithsonian Tropical Research Institute (STRI) Institutional Animal Care and Use Committee (IACUC) protocol 2017‐0101‐2020‐2.

## Conflicts of Interest

The authors declare no conflicts of interest.

## Supporting information


**Figure S1:** Distribution of IBD blocks along chromosomes for samples sequenced at the local scale in Belize (A), at the Greater Caribbean scale (B), and at the local scale again but with the TPBWT data (C). The two spatial scales and IBD calling methods show similar patterns. As expected, more IBD fragments are shared in regions of low recombination, including a putative inversion that covers a large part of chromosome 8. The red dotted line indicates the 95th percentile of the IBD block distribution.

**Figure S2:** *H. puella* density along the Mesoamerican Barrier Reef in Belize. At total of 43400m^2^ transect surveys were conducted from the North to the South (inset in Figure 1). The *H. puella* density is low and relatively constant along the barrier reef, with just a slight tendency to increase towards the South.

**Figure S3:** Distribution of IBD block length in cM. Panel A shows block lengths from the local and Greater Caribbean scale datasets, both estimated with TRUFFLE. Panel B compares the block length distribution obtained at the local scale with two different methods, TRUFFLE and TPBWT. Bins are 0.5 cM wide, except for the last one which includes all blocks larger than 5 cM.

**Figure S4:** Isolation by distance between pairs of individuals at the local scale in Belize, based on the IBD index without 95th percentile filter (left) and using an alternative approach for calling IBD blocks (templated positional Burrows‐Wheeler transform, right). The subtle pattern of isolation by distance is robust to these two methodological variations.

**Figure S5:** Estimated versus ground‐truthed mean effective dispersal rate across 120 simulated datasets.

**Table S1:** New *H. puella* genomes from Belize generated for the local scale analysis. Latitude and longitude given in decimal degrees and the date (YYYY‐MM‐DD) refer to sample collection. Depth reflects approximate sequencing depth after removing duplicate reads. Accession numbers indicates the sample record in the European Nucleotide Archive (ENA).
**Table S2:** *H. puella* genomes included in the Greater Caribbean scale analysis. Latitude and longitude given in decimal degrees and as the date (YYYY‐MM‐DD) refer to sample collection. Depth reflects approximate sequencing depth after removing duplicate reads. Accession numbers indicates the sample record in the European Nucleotide Archive (ENA).

## Data Availability

The genomic data have been uploaded in the European Nucleotide Archive (accession numbers provided in Tables S1 and S2). The scripts used for data analysis and simulations are available at https://github.com/currocam/tombers_etal.

## References

[mec70569-bib-0001] Aguillon, S. M. , J. W. Fitzpatrick , R. Bowman , et al. 2017. “Deconstructing Isolation‐By‐Distance: The Genomic Consequences of Limited Dispersal.” PLoS Genetics 13: e1006911. 10.1371/journal.pgen.1006911 PMC5542401 28771477

[mec70569-bib-0002] Al‐Asadi, H. , D. Petkova , M. Stephens , and J. Novembre . 2019. “Estimating Recent Migration and Population‐Size Surfaces.” PLoS Genetics 15: e1007908. 10.1371/journal.pgen.1007908 PMC6347299 30640906

[mec70569-bib-0003] Almany, G. R. , M. L. Berumen , S. R. Thorrold , S. Planes , and G. P. Jones . 2007. “Local Replenishment of Coral Reef Fish Populations in a Marine Reserve.” Science 316: 742–744. 10.1126/science.1140597 17478720

[mec70569-bib-0004] Atema, J. , G. Gerlach , and C. B. Paris . 2015. “Sensory Biology and Navigation Behavior of Reef Fish Larvae.” In Ecology of Fishes on Coral Reefs, 3–15. Cambridge University Press.

[mec70569-bib-0005] Baharian, S. , M. Barakatt , C. R. Gignoux , et al. 2016. “The Great Migration and African‐American Genomic Diversity.” PLoS Genetics 12: e1006059. 10.1371/journal.pgen.1006059 PMC4883799 27232753

[mec70569-bib-0006] Baumdicker, F. , G. Bisschop , D. Goldstein , et al. 2022. “Efficient Ancestry and Mutation Simulation With Msprime 1.0.” Genetics 220: iyab229. 10.1093/genetics/iyab229 PMC9176297 34897427

[mec70569-bib-0007] Benestan, L. , K. Fietz , N. Loiseau , et al. 2021. “Restricted Dispersal in a Sea of Gene Flow.” Proceedings of the Royal Society B 288: 20210458. 10.1098/rspb.2021.0458 PMC8131118 34004134

[mec70569-bib-0008] Bjelland, D. W. , U. Lingala , P. S. Patel , M. Jones , and M. C. Keller . 2017. “A Fast and Accurate Method for Detection of IBD Shared Haplotypes in Genome‐Wide SNP Data.” European Journal of Human Genetics 25: 617–624. 10.1038/ejhg.2017.6 PMC5437913 28176766

[mec70569-bib-0009] Broquet, T. , C. Johnson , E. Petit , I. Thompson , F. Burel , and J. Fryxell . 2006. “Dispersal and Genetic Structure in the American Marten, *Martes americana* .” Molecular Ecology 15: 1689–1697. 10.1111/j.1365-294X.2006.02878.x 16629821

[mec70569-bib-0012] Browning, S. R. , and B. L. Browning . 2012. “Identity by Descent Between Distant Relatives: Detection and Applications.” Annual Review of Genetics 46: 617–633. 10.1146/annurev-genet-110711-155534 22994355

[mec70569-bib-0013] Browning, S. R. , B. L. Browning , M. L. Daviglus , et al. 2018. “Ancestry‐Specific Recent Effective Population Size in the Americas.” PLoS Genetics 14: e1007385. 10.1371/journal.pgen.1007385 PMC5967706 29795556

[mec70569-bib-0014] Campuzano‐Jiménez, F. , A. Zwaenepoel , E. L. R. De Keyzer , and H. Svardal . 2026. “IdentityByDescentDispersal. Jl: Inferring Dispersal Rates With Identity‐By‐Descent Blocks.” Journal of Open Source Software 11: 9517.

[mec70569-bib-0015] Chevy, E. T. , J. Min , V. Caudill , et al. 2025. “Population Genetics Meets Ecology: A Guide to Individual‐Based Simulations in Continuous Landscapes.” Ecology and Evolution 15: e71098. 10.1002/ece3.71098 PMC11997375 40235724

[mec70569-bib-0016] Coulmance, F. , D. Akkaynak , Y. Le Poul , M. P. Höppner , W. O. McMillan , and O. Puebla . 2024. “Phenotypic and Genomic Dissection of Colour Pattern Variation in a Reef Fish Radiation.” Molecular Ecology 33: e17047. 10.1111/mec.17047 37337919

[mec70569-bib-0017] Danecek, P. , A. Auton , G. Abecasis , et al. 2011. “The Variant Call Format and VCFtools.” Bioinformatics 27: 2156–2158. 10.1093/bioinformatics/btr330 PMC3137218 21653522

[mec70569-bib-0018] Delaneau, O. , B. Howie , A. J. Cox , J.‐F. Zagury , and J. Marchini . 2013. “Haplotype Estimation Using Sequencing Reads.” American Journal of Human Genetics 93: 687–696. 10.1016/j.ajhg.2013.09.002 PMC3791270 24094745

[mec70569-bib-0019] Delaneau, O. , J.‐F. Zagury , and J. Marchini . 2013. “Improved Whole‐Chromosome Phasing for Disease and Population Genetic Studies.” Nature Methods 10: 5–6. 10.1038/nmeth.2307 23269371

[mec70569-bib-0020] DePristo, M. A. , E. Banks , R. Poplin , et al. 2011. “A Framework for Variation Discovery and Genotyping Using Next‐Generation DNA Sequencing Data.” Nature Genetics 43: 491–498. 10.1038/ng.806 PMC3083463 21478889

[mec70569-bib-0021] Dimitromanolakis, A. , A. D. Paterson , and L. Sun . 2019. “Fast and Accurate Shared Segment Detection and Relatedness Estimation in Un‐Phased Genetic Data via TRUFFLE.” American Journal of Human Genetics 105: 78–88. 10.1016/j.ajhg.2019.05.007 PMC6612710 31178127

[mec70569-bib-0022] Duranton, M. , F. Bonhomme , and P.‐A. Gagnaire . 2019. “The Spatial Scale of Dispersal Revealed by Admixture Tracts.” Evolutionary Applications 12: 1743–1756. 10.1111/eva.12829 PMC6752141 31548854

[mec70569-bib-0023] Freyman, W. A. , K. F. McManus , S. S. Shringarpure , E. M. Jewett , K. Bryc , and A. Auton . 2021. “Fast and Robust Identity‐By‐Descent Inference With the Templated Positional Burrows–Wheeler Transform.” Molecular Biology and Evolution 38: 2131–2151. 10.1093/molbev/msaa328 PMC8097300 33355662

[mec70569-bib-0024] Friedlaender, J. S. 1971. “Isolation by Distance in Bougainville.” Proceedings of the National Academy of Sciences 68: 704–707. 10.1073/pnas.68.4.704 PMC389024 5279511

[mec70569-bib-0025] Haller, B. C. , J. Galloway , J. Kelleher , P. W. Messer , and P. L. Ralph . 2019. “Tree‐Sequence Recording in SLiM Opens New Horizons for Forward‐Time Simulation of Whole Genomes.” Molecular Ecology Resources 19: 552–566. 10.1111/1755-0998.12968 PMC6393187 30565882

[mec70569-bib-0026] Haller, B. C. , P. L. Ralph , and P. W. Messer . 2026. “SLiM 5: Eco‐Evolutionary Simulations Across Multiple Chromosomes and Full Genomes.” Molecular Biology and Evolution 43: msaf313. 10.1093/molbev/msaf313 PMC12759289 41292177

[mec70569-bib-0027] Harris, K. , and R. Nielsen . 2013. “Inferring Demographic History From a Spectrum of Shared Haplotype Lengths.” PLoS Genetics 9: e1003521. 10.1371/journal.pgen.1003521 PMC3675002 23754952

[mec70569-bib-0028] Hauser, L. , and G. R. Carvalho . 2008. “Paradigm Shifts in Marine Fisheries Genetics: Ugly Hypotheses Slain by Beautiful Facts.” Fish and Fisheries 9: 333–362.

[mec70569-bib-0029] Helmkampf, M. , F. Coulmance , M. J. Heckwolf , et al. 2025. “Radiation With Reproductive Isolation in the Near‐Absence of Phylogenetic Signal.” Science Advances 11: eadt0973. 10.1126/sciadv.adt0973 PMC12292942 40712024

[mec70569-bib-0030] Hench, K. , M. Helmkampf , W. O. McMillan , and O. Puebla . 2022. “Rapid Radiation in a Highly Diverse Marine Environment.” Proceedings of the National Academy of Sciences of the United States of America 119: e2020457119. 10.1073/pnas.2020457119 PMC8794831 35042790

[mec70569-bib-0031] Hench, K. , M. Vargas , M. P. Höppner , W. O. McMillan , and O. Puebla . 2019. “Inter‐Chromosomal Coupling Between Vision and Pigmentation Genes During Genomic Divergence.” Nature Ecology & Evolution 3: 657–667. 10.1038/s41559-019-0814-5 30833758

[mec70569-bib-0032] Jones, G. P. , H. B. Harrison , M. L. Berumen , S. Planes , and S. R. Thorrold . 2022. “Larval Dispersal in Anemonefish Populations: Self‐Recruitment, Connectivity, and Metapopulation Dynamics.” In Evolution, Development and Ecology of Anemonefishes, 209–220. CRC Press.

[mec70569-bib-0033] Jones, G. P. , M. J. Milicich , M. J. Emslie , and C. Lunow . 1999. “Self‐Recruitment in a Coral Reef Fish Population.” Nature 402: 802–804.

[mec70569-bib-0034] Kimura, M. 1953. “’Stepping Stone'model of Population.” Annual Report of the National Institute of Genetics, Japan 3: 62–63.

[mec70569-bib-0035] Li, H. , and R. Durbin . 2009. “Fast and Accurate Short Read Alignment With Burrows–Wheeler Transform.” Bioinformatics 25: 1754–1760. 10.1093/bioinformatics/btp324 PMC2705234 19451168

[mec70569-bib-0036] Malécot, G. 1948. Les mathématiques de l'hérédité. Masson.

[mec70569-bib-0037] Manel, S. , N. Loiseau , M. Andrello , et al. 2019. “Long‐Distance Benefits of Marine Reserves: Myth or Reality?” Trends in Ecology & Evolution 34: 342–354. 10.1016/j.tree.2019.01.002 30777295

[mec70569-bib-0038] McCartney, M. A. , J. Acevedo , C. Heredia , et al. 2003. “Genetic Mosaic in a Marine Species Flock.” Molecular Ecology 12: 2963–2973. 10.1046/j.1365-294x.2003.01946.x 14629377

[mec70569-bib-0039] McKenna, A. , M. Hanna , E. Banks , et al. 2010. “The Genome Analysis Toolkit: A MapReduce Framework for Analyzing Next‐Generation DNA Sequencing Data.” Genome Research 20: 1297–1303. 10.1101/gr.107524.110 PMC2928508 20644199

[mec70569-bib-0040] Meirmans, P. G. 2012. “The Trouble With Isolation by Distance.” Molecular Ecology 21: 2839–2846. 10.1111/j.1365-294X.2012.05578.x 22574758

[mec70569-bib-0041] Naaykens, T. , and C. C. D'Aloia . 2022. “Isolation‐By‐Distance and Genetic Parentage Analysis Provide Similar Larval Dispersal Estimates.” Molecular Ecology 31: 3072–3082. 10.1111/mec.16465 35403317

[mec70569-bib-0042] Naseri, A. , X. Liu , K. Tang , S. Zhang , and D. Zhi . 2019. “RaPID: Ultra‐Fast, Powerful, and Accurate Detection of Segments Identical by Descent (IBD) in Biobank‐Scale Cohorts.” Genome Biology 20: 1–15. 10.1186/s13059-019-1754-8 PMC6659282 31345249

[mec70569-bib-0043] Palamara, P. F. , T. Lencz , A. Darvasi , and I. Pe'er . 2012. “Length Distributions of Identity by Descent Reveal Fine‐Scale Demographic History.” American Journal of Human Genetics 91: 809–822. 10.1016/j.ajhg.2012.08.030 PMC3487132 23103233

[mec70569-bib-0044] Palamara, P. F. , and I. Pe'er . 2013. “Inference of Historical Migration Rates via Haplotype Sharing.” Bioinformatics 29: i180–i188. 10.1093/bioinformatics/btt239 PMC3694674 23812983

[mec70569-bib-0045] Palumbi, S. R. , G. Grabowsky , T. Duda , L. Geyer , and N. Tachino . 1997. “Speciation and Population Genetic Structure in Tropical Pacific Sea Urchins.” Evolution 51: 1506–1517. 10.1111/j.1558-5646.1997.tb01474.x 28568622

[mec70569-bib-0046] Park, D. S. , Y. Baran , F. Hormozdiari , et al. 2015. “PIGS: Improved Estimates of Identity‐By‐Descent Probabilities by Probabilistic IBD Graph Sampling.” BMC Bioinformatics 16: 1–12. 10.1186/1471-2105-16-S5-S9 PMC4402697 25860540

[mec70569-bib-0047] Peñalba, J. V. , and J. B. Wolf . 2020. “From Molecules to Populations: Appreciating and Estimating Recombination Rate Variation.” Nature Reviews Genetics 21: 476–492. 10.1038/s41576-020-0240-1 32472059

[mec70569-bib-0048] Pinsky, M. L. , P. Saenz‐Agudelo , O. C. Salles , et al. 2017. “Marine Dispersal Scales Are Congruent Over Evolutionary and Ecological Time.” Current Biology 27: 149–154. 10.1016/j.cub.2016.10.053 27989671

[mec70569-bib-0049] Puebla, O. , A. Aguilar‐Perera , M. Helmkampf , et al. 2025. “Hypoplectrus Espinosai sp. Nov.(Teleostei: Serranidae), a New Hamlet on Coral Reefs in the Southwestern Gulf of Mexico.” Zootaxa 5618: 509–524. 10.11646/zootaxa.5618.4.3 41119429

[mec70569-bib-0050] Puebla, O. , E. Bermingham , and F. Guichard . 2009. “Estimating Dispersal From Genetic Isolation by Distance in a Coral Reef Fish ( *Hypoplectrus puella* ).” Ecology 90: 3087–3098. 10.1890/08-0859.1 19967864

[mec70569-bib-0051] Puebla, O. , E. Bermingham , and W. O. McMillan . 2012. “On the Spatial Scale of Dispersal in Coral Reef Fishes.” Molecular Ecology 21: 5675–5688. 10.1111/j.1365-294X.2012.05734.x 22994267

[mec70569-bib-0052] Puebla, O. , F. Coulmance , C. J. Estape , A. M. Estape , and D. R. Robertson . 2022. “A Review of 263 Years of Taxonomic Research on Hypoplectrus (Perciformes: Serranidae), With a Redescription of Hypoplectrus Affinis (Poey, 1861).” Zootaxa 5093: 101–141. 10.11646/zootaxa.5093.2.1 35390813

[mec70569-bib-0053] Ringbauer, H. , G. Coop , and N. H. Barton . 2017. “Inferring Recent Demography From Isolation by Distance of Long Shared Sequence Blocks.” Genetics 205: 1335–1351. 10.1534/genetics.116.196220 PMC5340342 28108588

[mec70569-bib-0054] Rousset . 2000. “Genetic Differentiation Between Individuals.” Journal of Evolutionary Biology 13: 58–62.

[mec70569-bib-0055] Rousset, F. 1997. “Genetic Differentiation and Estimation of Gene Flow From F‐Statistics Under Isolation by Distance.” Genetics 145: 1219–1228. 10.1093/genetics/145.4.1219 PMC1207888 9093870

[mec70569-bib-0056] Seidman, D. N. , S. A. Shenoy , M. Kim , et al. 2020. “Rapid, Phase‐Free Detection of Long Identity‐By‐Descent Segments Enables Effective Relationship Classification.” American Journal of Human Genetics 106: 453–466. 10.1016/j.ajhg.2020.02.012 PMC7118564 32197076

[mec70569-bib-0057] Sumner, J. , F. Rousset , A. Estoup , and C. Moritz . 2001. “Neighbourhood'size, Dispersal and Density Estimates in the Prickly Forest Skink (Gnypetoscincus Queenslandiae) Using Individual Genetic and Demographic Methods.” Molecular Ecology 10: 1917–1927. 10.1046/j.0962-1083.2001.01337.x 11555236

[mec70569-bib-0058] Swearer, S. E. , J. E. Caselle , D. W. Lea , and R. R. Warner . 1999. “Larval Retention and Recruitment in an Island Population of a Coral‐Reef Fish.” Nature 402: 799–802.

[mec70569-bib-0059] Theodosiou, L. , W. McMillan , and O. Puebla . 2016. “Recombination in the Eggs and Sperm in a Simultaneously Hermaphroditic Vertebrate.” Proceedings of the Royal Society B: Biological Sciences 283: 20161821. 10.1098/rspb.2016.1821 PMC5204151 27974520

[mec70569-bib-0060] Twyford, A. D. , E. L. Wong , and J. Friedman . 2020. “Multi‐Level Patterns of Genetic Structure and Isolation by Distance in the Widespread Plant *Mimulus guttatus* .” Heredity 125: 227–239. 10.1038/s41437-020-0335-7 PMC7490260 32641721

[mec70569-bib-0061] Van der Auwera, G. A. , M. O. Carneiro , C. Hartl , et al. 2013. “From FastQ Data to High‐Confidence Variant Calls: The Genome Analysis Toolkit Best Practices Pipeline.” Current Protocols in Bioinformatics 43: 11. 10.1002/0471250953.bi1110s43 PMC4243306 25431634

[mec70569-bib-0062] Vos, M. , and G. J. Velicer . 2008. “Isolation by Distance in the Spore‐Forming Soil Bacterium *Myxococcus xanthus* .” Current Biology 18: 386–391. 10.1016/j.cub.2008.02.050 18328701

[mec70569-bib-0063] Watts, P. C. , F. Rousset , I. J. Saccheri , R. Leblois , S. J. Kemp , and D. J. Thompson . 2007. “Compatible Genetic and Ecological Estimates of Dispersal Rates in Insect (Coenagrion Mercuriale: Odonata: Zygoptera) Populations: Analysis of ‘Neighbourhood Size'using a More Precise Estimator.” Molecular Ecology 16: 737–751. 10.1111/j.1365-294X.2006.03184.x 17284208

[mec70569-bib-0064] Wirth, T. , and L. Bernatchez . 2001. “Genetic Evidence Against Panmixia in the European Eel.” Nature 409: 1037–1040. 10.1038/35059079 11234011

[mec70569-bib-0065] Wright, S. 1931. “Evolution in Mendelian Populations.” Genetics 16: 97–159. 10.1093/genetics/16.2.97 PMC1201091 17246615

[mec70569-bib-0066] Wright, S. 1938. “Size of Population and Breeding Structure in Relation to Evolution.” Science 87: 430–431.

[mec70569-bib-0067] Wright, S. 1940. “Breeding Structure of Populations in Relation to Speciation.” American Naturalist 74: 232–248.

[mec70569-bib-0068] Wright, S. 1943. “Isolation by Distance.” Genetics 28: 114–138. 10.1093/genetics/28.2.114 PMC1209196 17247074

[mec70569-bib-0069] Yang, J. , B. Benyamin , B. P. McEvoy , et al. 2010. “Common SNPs Explain a Large Proportion of the Heritability for Human Height.” Nature Genetics 42: 565–569. 10.1038/ng.608 PMC3232052 20562875

[mec70569-bib-0070] Zhou, Y. , S. R. Browning , and B. L. Browning . 2020. “A Fast and Simple Method for Detecting Identity‐By‐Descent Segments in Large‐Scale Data.” American Journal of Human Genetics 106: 426–437. 10.1016/j.ajhg.2020.02.010 PMC7118582 32169169

