# Supplementary material for: Blocks of Identity by Descent Reveal the Dual Role Played by Reef Fish Larvae for Retention and Dispersal
Source: Mol Ecol. 2026 Sep 28;35(19):e70569. doi: 10.1111/mec.70569 (PMC13619822; doi:10.1111/mec.70569)
Supplement: Supplementary file 1 [file MEC-35-e70569-s001.pdf]

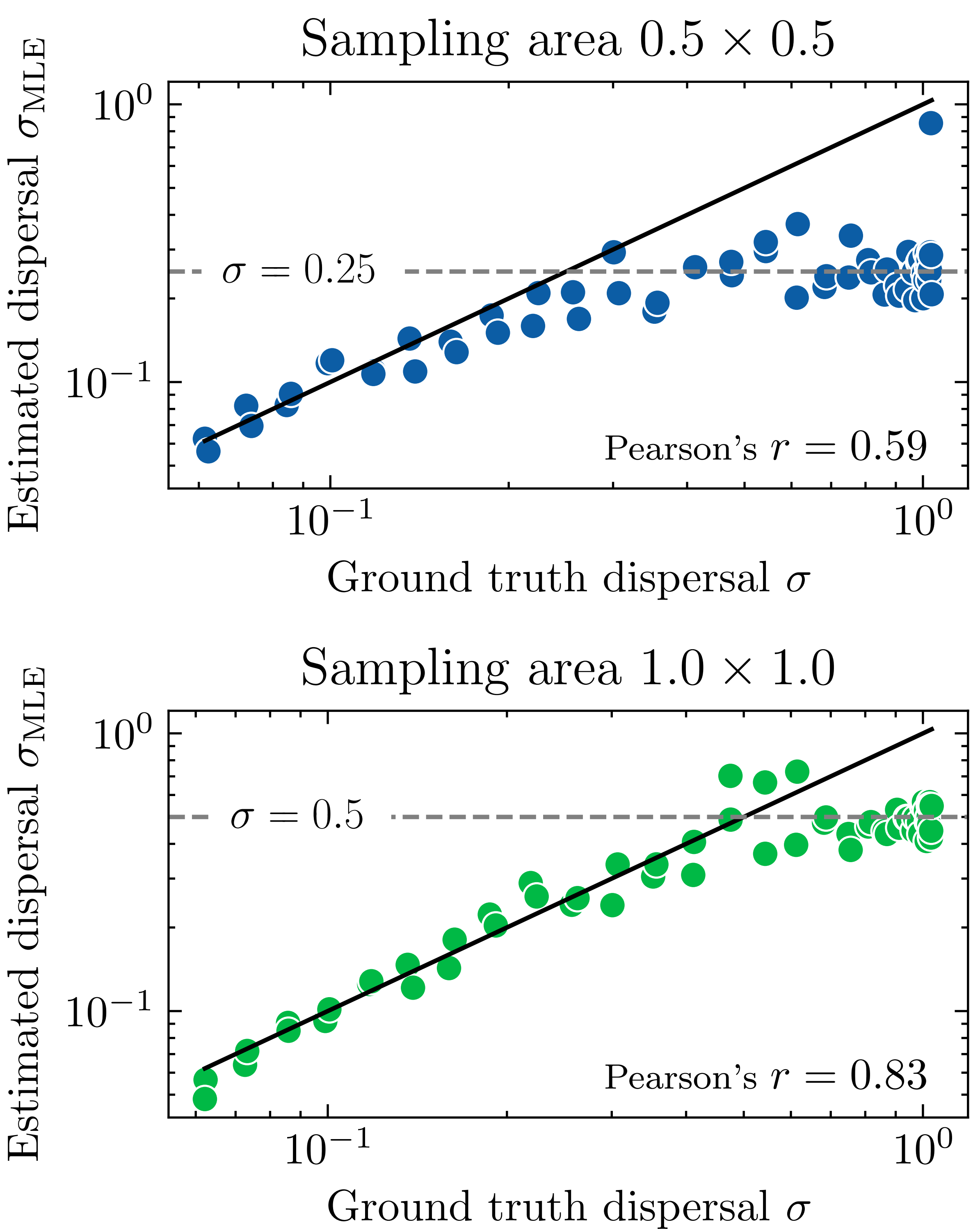
Sampling area 0.5 × 0.5
Estimated dispersal σ_{MLE}
10^0
10^{-1}
$\sigma = 0.25$
Pearson's $r = 0.59$
10^{-1}
10^0
Ground truth dispersal σ
Sampling area 1.0 × 1.0
Estimated dispersal σ_{MLE}
10^0
10^{-1}
$\sigma = 0.5$
Pearson's $r = 0.83$
10^{-1}
10^0
Ground truth dispersal σ
