# Supplementary material for: Blocks of Identity by Descent Reveal the Dual Role Played by Reef Fish Larvae for Retention and Dispersal
Source: Mol Ecol. 2026 Sep 28;35(19):e70569. doi: 10.1111/mec.70569 (PMC13619822; doi:10.1111/mec.70569)
Supplement: Supplementary file 2 [file MEC-35-e70569-s002.pdf]

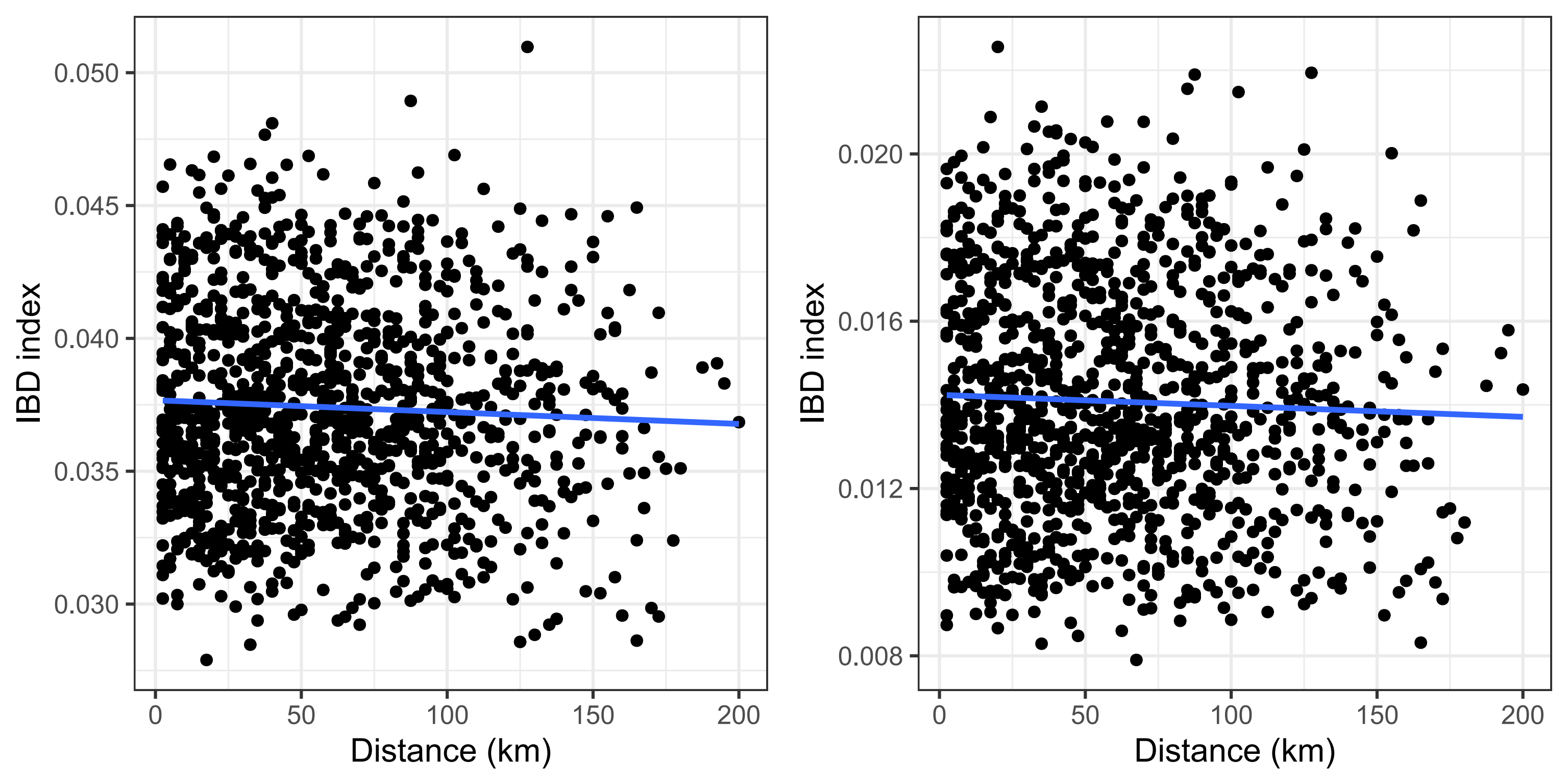
0.050
0.045
0.040
0.035
0.030
IBD index
0
50
100
150
200
Distance (km)
0.020
0.016
0.012
0.008
IBD index
0
50
100
150
200
Distance (km)
