# Supplementary material for: Blocks of Identity by Descent Reveal the Dual Role Played by Reef Fish Larvae for Retention and Dispersal
Source: Mol Ecol. 2026 Sep 28;35(19):e70569. doi: 10.1111/mec.70569 (PMC13619822; doi:10.1111/mec.70569)
Supplement: Supplementary file 3 [file MEC-35-e70569-s003.pdf]

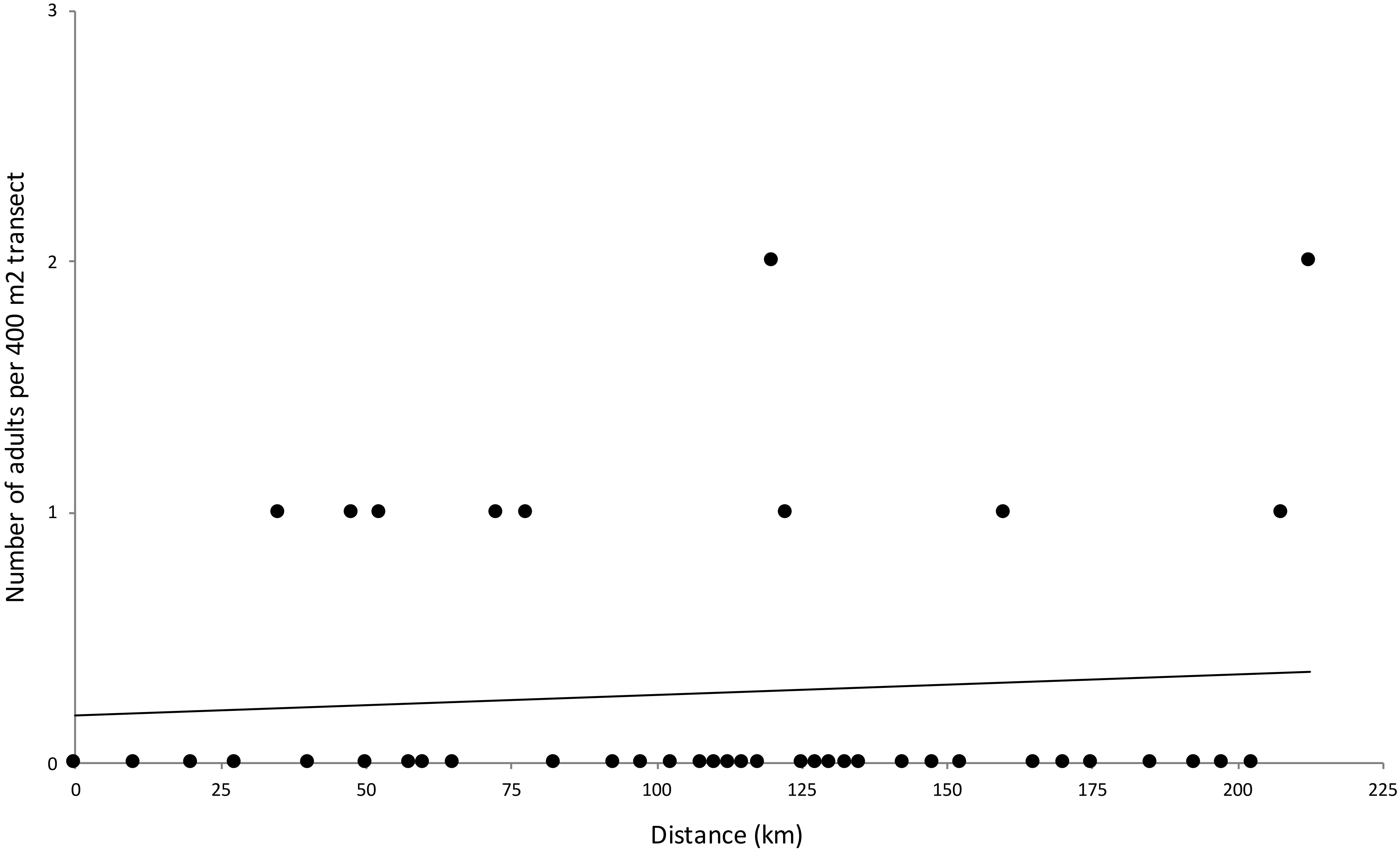

Number of adults per 400 m2 transect
3
2
1
0
0
25
50
75
100
125
150
175
200
225
Distance (km)
