# Supplementary material for: Blocks of Identity by Descent Reveal the Dual Role Played by Reef Fish Larvae for Retention and Dispersal
Source: Mol Ecol. 2026 Sep 28;35(19):e70569. doi: 10.1111/mec.70569 (PMC13619822; doi:10.1111/mec.70569)
Supplement: Supplementary file 4 [file MEC-35-e70569-s004.pdf]

TABLE S1 New *H. puella* genomes from Belize generated for the local scale analysis. Latitude and longitude given in decimal degrees and the date (YYYY-MM-DD) refer to sample collection. Depth reflects approximate sequencing depth after removing duplicate reads. Accession numbers indicates the sample record in the European Nucleotide Archive (ENA)

SampleID	Location	Latitude	Longitude	Date	Depth	Accession
24222	Belize	17.74913	-87.99505	2010-09-06	20.5	ERS30347991
24230	Belize	17.57360	-88.03898	2010-09-06	17.4	ERS30347992
24231	Belize	17.55238	-88.04198	2010-09-06	21.0	ERS30347993
24233	Belize	17.50750	-88.04578	2010-09-06	20.2	ERS30347994
24235	Belize	17.46362	-88.03397	2010-09-06	24.6	ERS30347995
24236	Belize	17.44122	-88.03180	2010-09-06	25.8	ERS30347996
24237	Belize	17.41875	-88.03180	2010-09-06	21.9	ERS30347997
24238	Belize	17.39600	-88.03352	2010-09-06	17.8	ERS30347998
24239	Belize	17.37357	-88.03537	2010-09-06	19.5	ERS30347999
24241	Belize	17.33255	-88.02025	2010-09-06	29.4	ERS30348000
24242	Belize	17.31522	-88.03503	2010-09-06	18.9	ERS30348001
24243	Belize	17.29232	-88.03817	2010-09-06	20.3	ERS30348002
24244	Belize	17.26973	-88.04467	2010-09-06	26.6	ERS30348003
24245	Belize	17.24825	-88.04483	2010-09-06	31.0	ERS30348004
24246	Belize	17.22658	-88.04438	2010-09-06	23.1	ERS30348005
24247	Belize	17.20323	-88.05065	2010-09-06	33.1	ERS30348006
24248	Belize	17.18082	-88.04963	2010-09-06	28.4	ERS30348007
24249	Belize	17.15838	-88.04690	2010-09-06	33.1	ERS30348008
24250	Belize	17.13713	-88.03898	2010-09-06	18.2	ERS30348009
24251	Belize	17.12087	-88.02685	2010-09-06	26.6	ERS30348010
24256	Belize	17.01998	-88.00003	2010-09-06	22.5	ERS30348011
24259	Belize	16.95877	-88.03800	2010-09-06	22.0	ERS30348012
24260	Belize	16.93628	-88.04058	2010-09-06	32.4	ERS30348013
24261	Belize	16.91480	-88.04822	2010-09-06	26.2	ERS30348014
24262	Belize	16.89355	-88.05672	2010-09-06	26.2	ERS30348015
24263	Belize	16.87230	-88.06362	2010-09-06	30.8	ERS30348016
24264	Belize	16.85037	-88.07012	2010-09-06	25.7	ERS30348017
24265	Belize	16.82813	-88.07508	2010-09-06	24.7	ERS30348018
24266	Belize	16.80590	-88.07685	2010-09-06	29.4	ERS30348019
24267	Belize	16.78332	-88.07543	2010-09-06	31.8	ERS30348020
24269	Belize	16.73813	-88.07260	2010-09-06	19.0	ERS30348021
24270	Belize	16.71512	-88.07187	2010-09-06	24.4	ERS30348022

continued on next page

SampleID	Location	Latitude	Longitude	Date	Depth	Accession
24271	Belize	16.69167	-88.07083	2010-09-06	25.2	ERS30348023
24272	Belize	16.67167	-88.06667	2010-09-06	24.9	ERS30348024
24273	Belize	16.65000	-88.05833	2010-09-06	24.6	ERS30348025
24274	Belize	16.62500	-88.05250	2010-09-06	25.0	ERS30348026
24275	Belize	16.60500	-88.03833	2010-09-06	26.9	ERS30348027
24276	Belize	16.58750	-88.02500	2010-09-06	25.4	ERS30348028
24281	Belize	16.48500	-87.97917	2010-09-06	19.8	ERS30348029
24282	Belize	16.46667	-87.99500	2010-09-06	29.5	ERS30348030
24283	Belize	16.45000	-88.01333	2010-09-06	25.1	ERS30348031
24284	Belize	16.42917	-88.02500	2010-09-06	29.4	ERS30348032
24285	Belize	16.41250	-88.03750	2010-09-06	23.8	ERS30348033
24286	Belize	16.39583	-88.05833	2010-09-06	23.1	ERS30348034
24290	Belize	16.32083	-88.12333	2010-09-06	21.5	ERS30348035
24297	Belize	16.17917	-88.21417	2010-09-06	19.9	ERS30348036
24299	Belize	16.13750	-88.23667	2010-09-06	31.9	ERS30348037
24300	Belize	16.11667	-88.25000	2010-09-06	24.4	ERS30348038
24202	Belize	16.07917	-88.28333	2010-09-06	19.4	ERS30347990

TABLE S2 *H. puella* genomes included in the Greater Caribbean scale analysis. Latitude and longitude given in decimal degrees and as the date (YYYY-MM-DD) refer to sample collection. Depth reflects approximate sequencing depth after removing duplicate reads. Accession numbers indicates the sample record in the European Nucleotide Archive (ENA)

SampleID	Location	Latitude	Longitude	Date	Depth	Accession
18419	Bocas del Toro, Panama	9.33278	-83.17306	2004-05-12	16.8	ERS2619661
18422	Bocas del Toro, Panama	9.37750	-82.30389	2004-05-12	18.8	ERS2619663
18426	Bocas del Toro, Panama	9.33278	-83.17306	2004-05-12	17.1	ERS2619664
18427	Bocas del Toro, Panama	9.33278	-83.17306	2004-05-12	17.5	ERS2619665
18429	Bocas del Toro, Panama	9.33278	-83.17306	2004-05-12	20.0	ERS2619666
18430	Bocas del Toro, Panama	9.33278	-83.17306	2004-05-12	16.8	ERS2619667
18432	Bocas del Toro, Panama	9.33278	-83.17306	2004-05-12	18.6	ERS2619668
18434	Bocas del Toro, Panama	9.37750	-82.30389	2004-05-12	23.1	ERS2619669
18912	Bocas del Toro, Panama	9.29833	-82.28944	2005-03-25	20.6	ERS2619670
18917	Bocas del Toro, Panama	9.29833	-82.28944	2005-03-25	16.3	ERS2619672
19104	Guna Yala, Panama	9.57592	-78.72794	2005-05-08	37.9	ERS20924432
19106	Guna Yala, Panama	9.57592	-78.72794	2005-05-08	24.9	ERS20924434
19108	Guna Yala, Panama	9.57592	-78.72794	2005-05-08	29.6	ERS20924435
19426	Barbados	9.33278	-59.64506	2005-06-06	30.0	ERS20924446
19427	Barbados	9.33278	-59.64506	2005-06-06	22.0	ERS20924447
19428	Barbados	9.33278	-59.64506	2005-06-06	18.9	ERS20924448
20551	Honduras	9.33278	-83.29306	2006-06-04	17.9	ERS2619649
20553	Honduras	9.33278	-83.29306	2006-06-04	19.6	ERS2619651
20554	Honduras	9.33278	-83.29306	2006-06-04	17.2	ERS2619652
20556	Honduras	9.33278	-83.29306	2006-06-04	21.6	ERS2619654
20558	Honduras	9.33278	-83.29306	2006-06-04	19.5	ERS2619655
20559	Honduras	9.33278	-83.29306	2006-06-04	20.8	ERS2619656
20625	Honduras	9.33278	-83.29306	2006-06-05	16.6	ERS2619657
20633	Honduras	9.33278	-83.29306	2006-06-05	21.1	ERS2619658
20635	Honduras	9.33278	-83.29306	2006-06-05	20.2	ERS2619659
20638	Honduras	9.33278	-83.29306	2006-06-05	16.3	ERS2619660
24235	Belize	17.74913	-87.99505	2010-09-06	24.6	ERS30347995
24242	Belize	17.74913	-87.99505	2010-09-06	18.9	ERS30348001
24247	Belize	17.74913	-87.99505	2010-09-06	33.1	ERS30348006
24261	Belize	17.74913	-87.99505	2010-09-06	26.2	ERS30348014
24281	Belize	17.74913	-87.99505	2010-09-06	19.8	ERS30348029
24282	Belize	17.74913	-87.99505	2010-09-06	29.5	ERS30348030

continued on next page

SampleID	Location	Latitude	Longitude	Date	Depth	Accession
24283	Belize	17.74913	-87.99505	2010-09-06	25.1	ERS30348031
24284	Belize	17.74913	-87.99505	2010-09-06	29.4	ERS30348032
24285	Belize	17.74913	-87.99505	2010-09-06	23.8	ERS30348033
24300	Belize	17.74913	-87.99505	2010-09-06	24.4	ERS30348038
PL17_139	Florida Keys, USA	9.33278	-80.67677	2017-08-07	29.5	ERS14948450
PL17_155	Florida Keys, USA	9.33278	-80.36930	2017-07-14	29.6	ERS14948449
PL17_157	Florida Keys, USA	9.33278	-80.83116	2017-07-15	24.9	ERS14948446
PL17_159	Florida Keys, USA	9.33278	-80.92229	2017-07-16	26.7	ERS14948445
PL17_35	Puerto Rico	9.33278	-67.05643	2017-03-15	28.5	ERS14948482
PL17_50	Puerto Rico	9.33278	-67.05325	2017-03-16	24.5	ERS14948475
PL17_53	Puerto Rico	9.33278	-67.05325	2017-03-16	22.1	ERS14948476
PL17_54	Puerto Rico	9.33278	-67.05325	2017-03-16	28.5	ERS14948483
PL17_57	Puerto Rico	9.33278	-67.05804	2017-03-17	28.4	ERS14948479
PL17_60	Puerto Rico	9.33278	-67.05804	2017-03-17	30.4	ERS14948481
PL17_65	Puerto Rico	9.33278	-67.04642	2017-03-18	26.1	ERS14948490
PL17_69	Puerto Rico	9.33278	-67.01714	2017-03-21	28.6	ERS14948486
PL17_82	Puerto Rico	unknown	unknown	2017-03-23	37.0	ERS14948473
