# Supplementary material for: Blocks of Identity by Descent Reveal the Dual Role Played by Reef Fish Larvae for Retention and Dispersal
Source: Mol Ecol. 2026 Sep 28;35(19):e70569. doi: 10.1111/mec.70569 (PMC13619822; doi:10.1111/mec.70569)
Supplement: Supplementary file 5 [file MEC-35-e70569-s005.pdf]

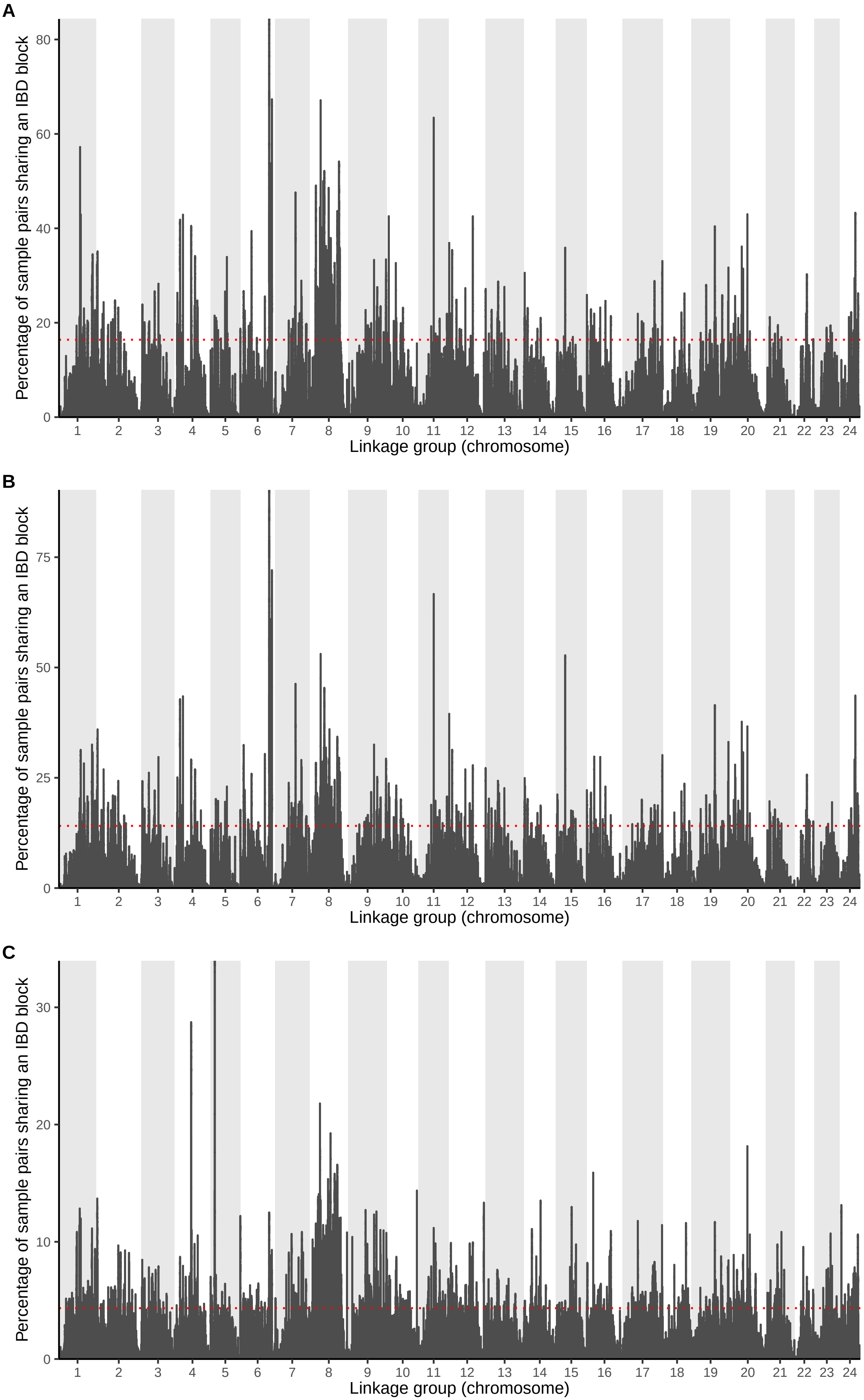
A
Percentage of sample pairs sharing an IBD block
Linkage group (chromosome)
B
Percentage of sample pairs sharing an IBD block
Linkage group (chromosome)
C
Percentage of sample pairs sharing an IBD block
Linkage group (chromosome)
