# Supplementary material for: Blocks of Identity by Descent Reveal the Dual Role Played by Reef Fish Larvae for Retention and Dispersal
Source: Mol Ecol. 2026 Sep 28;35(19):e70569. doi: 10.1111/mec.70569 (PMC13619822; doi:10.1111/mec.70569)
Supplement: Supplementary file 6 [file MEC-35-e70569-s006.pdf]

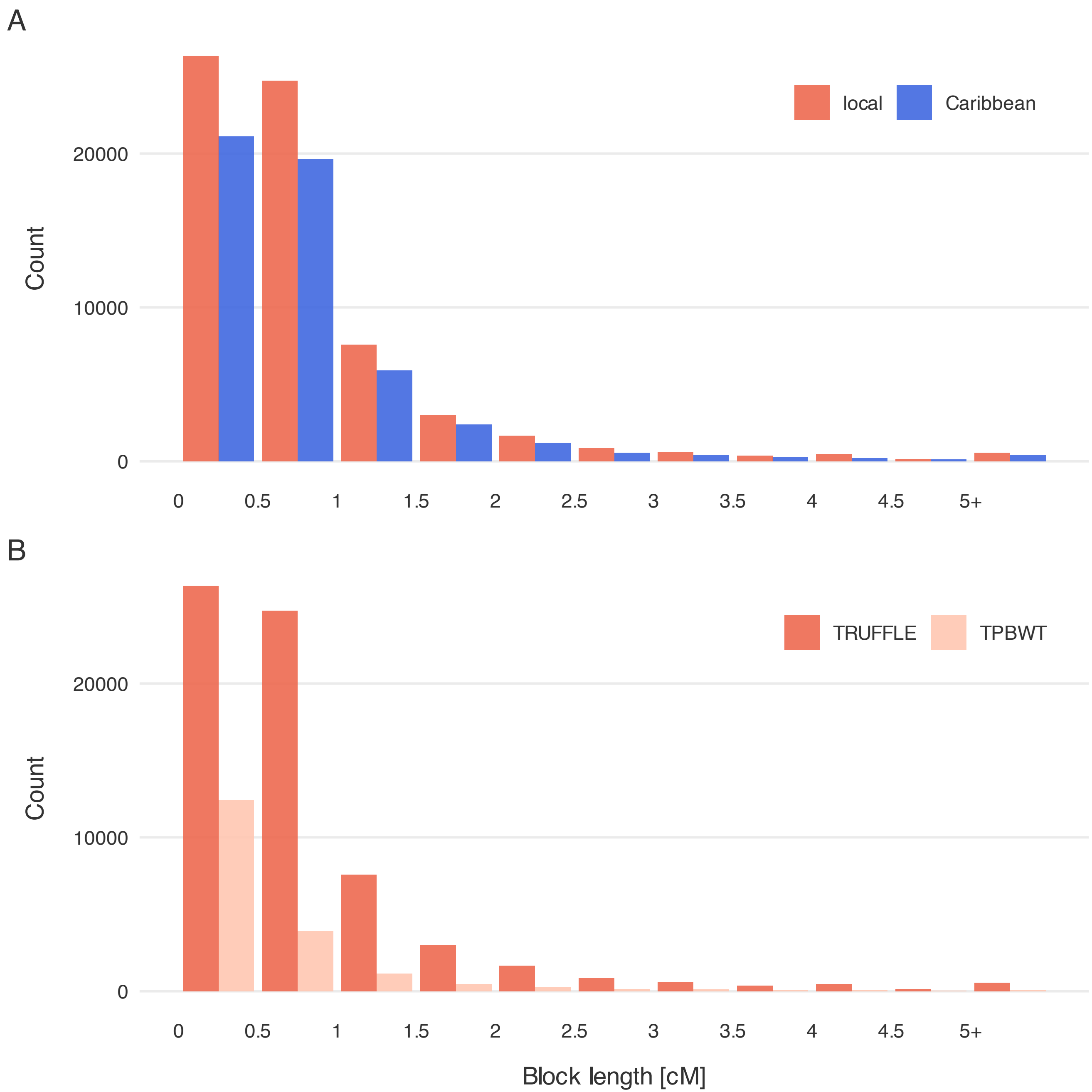
A
local
Caribbean
20000
10000
0
Count
0
0.5
1
1.5
2
2.5
3
3.5
4
4.5
5+
B
TRUFFLE
TPBWT
20000
10000
0
Count
0
0.5
1
1.5
2
2.5
3
3.5
4
4.5
5+
Block length [cM]
